# Feasibility of a 12 Week Physical Intervention to Prevent Cognitive Decline and Disability in the At-Risk Elderly Population in Korea

**DOI:** 10.3390/jcm9103135

**Published:** 2020-09-28

**Authors:** Sun Min Lee, Hong-sun Song, Buong-O Chun, Muncheong Choi, Kyunghwa Sun, Ki Sub Kim, Hyesu Jeon, Da Eun Seo, Hye Mi Kwon, Jee Hyang Jeong, Yoo Kyoung Park, Chang Hyung Hong, Hae Ri Na, Seong Hye Choi, So Young Moon

**Affiliations:** 1 Department of Neurology, Ajou University School of Medicine, Suwon 16499, Korea; casicielo9@gmail.com (S.M.L.); nabear1001@naver.com (K.S.); sunlee@ajou.ac.kr (H.M.K.); 2 Department of Medical Sciences, Graduate School of Ajou University, Suwon 16499, Korea; 3 Department of Sports Science, Korea Institute of Sport Science, Seoul 01794, Korea; hssong@kspo.or.kr (H.-s.S.); tianbingwu@naver.com (B.-O.C.); 4 Exercowork, Hanam 12912, Korea; bdforever@hanmail.net; 5 Dongtan Public Health Center, Hwaseong 18460, Korea; sunlee@aumc.ac.kr; 6 Department of Psychology, Ajou University, Suwon 16499, Korea; dapin1203@daum.net (H.J.); daeuneee_711@naver.com (D.E.S.); 7 Department of Neurology, Ewha Womans University School of medicine, Seoul 07804, Korea; jjeong@ewha.ac.kr; 8 Department of Medical Nutrition, Graduate School of East-West Medical Nutrition, Kyung Hee University, Suwon 17104, Korea; ypark@khu.ac.kr; 9 Department of Psychiatry, Ajou University School of Medicine, Suwon 16499, Korea; chhong2012@gmail.com; 10 Department of Neurology, Bobath Memorial Hospital, Seongnam 13552, Korea; neuna102@paran.com; 11 Department of Neurology, Inha University College of Medicine, Incheon 22212, Korea; seonghye@inha.ac.kr

**Keywords:** dementia prevention, cognitive decline, disability, physical fitness, physical exercise intervention, feasibility, safety

## Abstract

There is a need for measures that can prevent the onset of dementia in the rapidly aging population. Reportedly, sustained physical exercise can prevent cognitive decline and disability. This study aimed to assess the feasibility of a 12-week physical exercise intervention (PEI) for delay of cognitive decline and disability in the at-risk elderly population in Korea. Twenty-six participants (aged 67.9 ± 3.6 years, 84.6% female) at risk of dementia were assigned to facility-based PEI (*n* = 15) or home-based PEI (*n* = 11). The PEI program consisted of muscle strength training, aerobic exercise, balance, and stretching using portable aids. Feasibility was assessed by retention and adherence rates. Physical fitness/cognitive function were compared before and after the PEI. Retention and adherence rates were 86.7% and 88.3%, respectively, for facility-based PEI and 81.8% and 62.3% for home-based PEI. No intervention-related adverse events were reported. Leg strength/endurance and cardiopulmonary endurance were improved in both groups: 30 s sit-to-stand test (facility-based, *p* = 0.002; home-based, *p* = 0.002) and 2 -min stationary march (facility-based, *p* = 0.001; home-based, *p* = 0.022). Cognitive function was improved only after facility-based PEI (Alzheimer’s Disease Assessment Scale-cognitive total score, *p* = 0.009; story memory test on Literacy Independent Cognitive Assessment, *p* = 0.026). We found that, whereas our PEI is feasible, the home-based program needs supplementation to improve adherence.

## 1. Introduction

South Korea has one of the most rapidly aging populations in the world, and dementia has emerged as a major health problem in this country [1]. Therefore, there is an urgent need for measures that can delay or prevent the onset of dementia in South Korea. Several recent studies in Western countries suggest that multidomain lifestyle intervention, including dietary counseling, physical exercise, cognitive training, and vascular and metabolic risk monitoring, can delay cognitive decline and progression to dementia in at-risk individuals [2,3,4,5,6,7,8,9,10]. A dementia prevention program targeting the at-risk elderly, termed SUPERBRAIN (SoUth Korean study to PrEvent cognitive impaiRment and protect BRAIN), is being developed with the support of the Ministry of Health and Welfare in South Korea [11]. This program consists of management of metabolic and vascular risk factors, cognitive training and social activity, physical exercise, nutritional guidance, and motivational enhancement strategies. It includes both facility-based and home-based multidomain intervention programs suitable for elderly Koreans and is a modified version of the FINGER (the Finnish Geriatric Intervention Study to Prevent Cognitive Impairment and Disability) strategy [7].

Sustained physical exercise can prevent cognitive decline and disability [12]. Hence, facility-based physical exercise intervention (PEI) and home-based PEI programs that are tailored to the cultural and physical characteristics of the elderly population in Korea were developed as a component of SUPERBRAIN. Many Korean elderly individuals attend public health centers or senior citizens’ welfare centers for leisure activities or walk around their homes instead of using fitness centers. Therefore, exercise programs that can be implemented at public facilities at the home are needed. A recent study found that moderate-to-severe obesity was not uncommon among elderly men in Korea, cortical thinning in the frontal and temporal regions was significantly greater in underweight individuals than in their counterparts with normal weight, and overweight (mild obesity) was associated with increased cortical thickness [13]. Therefore, the emphasis has shifted to developing a more appropriate PEI for Korean elderly individuals that balances various modes of exercise, including aerobic, resistance, balance, and stretching, rather than focusing only on aerobic exercises.

In this study, we assessed the feasibility of facility-based and home-based PEI programs before their incorporation into SUPERBRAIN to prevent cognitive decline and disability in the at-risk elderly population in Korea. The hypothesis tested was that adherence and retention rates would be at least 75% in both facility-based PEI and home-based PEI.

## 2. Materials and Methods

### 2.1. Participants

Individuals aged 65–79 years were recruited from a community health center in Dongtan, Gyeonggi-do, South Korea, to participate in this feasibility trial of a 12 week PEI program that ran from September 2018 to December 2018. The inclusion criterion was not having dementia but being at risk of dementia with at least one of the following risk factors: a confirmed diagnosis of hypertension, diabetes mellitus, dyslipidemia, or obesity (body mass index [BMI] ≥25); lifetime smoking of at least 100 cigarettes or smoking of more than one cigarette in the past month; consumption of ≥170 g of alcohol per week; <9 years of formal education; and <150 min of moderate-to-vigorous activity and/or <75 min of vigorous activity per week. The exclusion criteria were a confirmed psychiatric diagnosis (e.g., major depressive disorder), dementia, other neurodegenerative disease (e.g., Parkinson’s disease), severe or unstable symptomatic cardiovascular disease, a diagnosis of incurable malignancy within the previous 5 years, angioplasty or a stent procedure within the previous year, a z-score below −1.5 on the Korean version of Mini-Mental State Examination (K-MMSE) [14], any other evidence of a severe or unstable physical condition, severe visual or hearing loss or communication impairment beyond which validation of intervention could not be performed, illiteracy, inability to participate safely and fully in the study in the opinion of the investigators, and involvement in other interventional studies. The participants were assigned to facility-based PEI or home-based PEI according to their preference. The trial was registered at the Clinical Research Information Service (KCT0003513) and approved by the Ajou University Hospital institutional review board (IRB) (AJIRB-BMR-SUR-18-277). Protocol modifications were reported to and approved by the IRB. Written informed consent was obtained from all potential participants before they were enrolled in the study.

### 2.2. Assessment of Physical Activity

During screening for enrollment, each potential participant’s level of physical activity was assessed using the Global Physical Activity Questionnaire (GPAQ) [15], whereby minutes per week spent in moderate-to-vigorous physical activity (MVPA) in various domains (work, transport, leisure) were calculated and reported. The total MVPA was then calculated by adding the three domains of activity. Compliance with World Health Organization physical activity guidelines [16] was assessed by classifying participants who performed 150 min of moderate or vigorous activity per week and/or 75 min of vigorous activity per week as “active” and those who did not meet these guidelines as “inactive”.

### 2.3. Assessment of Physical Performances

Before the intervention, physical performance was evaluated by sports science experts using the physical fitness test battery for the elderly in the Korean National Physical Performance Evaluation Program, which was developed by the national project of the Ministry of Cultures, Sports and Tourism of Korea. Items in the battery were developed to assess the level of physical performance of elderly individuals aged 65 or older, and the criteria for assessment of physical fitness by sex and age have been validated [17,18]. The physical fitness battery consists of two parts. The first includes three physical items (height, weight, and BMI). The second includes six physical fitness items for testing strength, flexibility, coordination, and balance as well as cardiopulmonary endurance: the hand grip strength test (kg, upper extremity strength), 30 s sit-to-stand test (times/30 s, leg strength/endurance), 3 m sit–walk-and-return test (seconds, balance), sit-and-reach test (cm, flexibility), 2 min stationary march (times/2 min, cardiopulmonary endurance), and Figure-8-walks (seconds, coordination). Poor physical fitness was defined as physical performance in any test being recorded as below the 30th percentile of the norms for the normal population matched for age and sex.

### 2.4. PEI Protocols

The PEI program consists of aerobic exercise, muscle strength training, postural balance, and flexibility exercises (Table 1). The intensity and difficulty level of physical exercise was increased every 4 weeks according to the structured protocol. All PEI was carried out using floor plates on which numbers (0 to 9) were drawn, elastic bands, immobile chairs, and cordless jump ropes. A detailed description of the components of each type of movement included at each level is provided in Appendix A.

**Figure 1:**
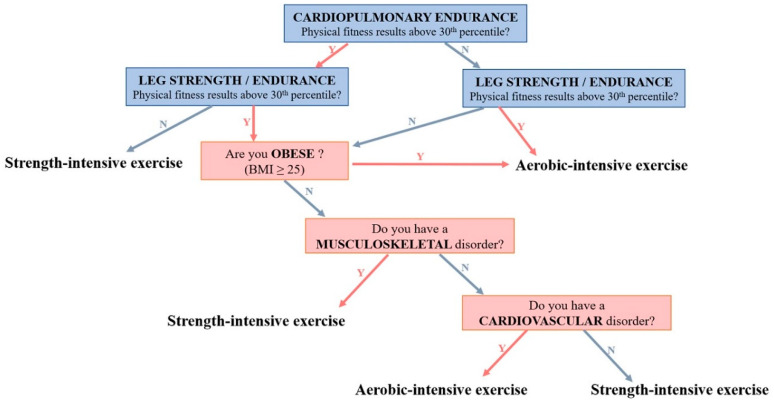
Algorithm for recommendation of exercise types based on physical performance and medical conditions. Cardiopulmonary endurance and leg strength/endurance were determined by the records of 2 min walk steps and sit-to-stand for 30 s, respectively. Poor physical fitness was defined as the recorded physical performance in any test being below the 30th percentile of the norms for the normal population matched for age and sex.

Participants allocated to home-based PEI were instructed to perform a strength-intensive or an aerobic-intensive exercise program based on their physical performance and physical condition (Figure 1). The strength-intensive program included 25 min of muscle strengthening exercise and 20 min of aerobic exercise in each session, whereas the aerobic-intensive program included 25 min of aerobic exercise and 20 min of muscle strengthening exercise in each session. Details of the algorithm used to assign each participant to the type of exercise program are provided in Figure 1. The aerobic-intensive program was recommended for individuals with poor cardiopulmonary endurance, BMI ≥ 25, or a cardiovascular disorder. Participants with poor muscular strength/endurance or a musculoskeletal disorder were assigned to the strength-intensive program.

Participants who opted for facility-based PEI visited the community center three times a week and undertook a 60 min exercise session at each visit. Facility-based PEI was performed in a group and guided by two trained exercise professionals. One exercise professional put a command in front of the group and demonstrated the movement, while the other moved between participants, correcting movements, and checking for safety. The home-based PEI consisted of a weekly 60 min group workout at the community center, and two sessions were held separately according to whether the program was strength-intensive or aerobic exercise-intensive. Whenever participants in the home-based PEI attended the group session, exercise professionals provided tips on how to incorporate the exercises at home. The participants exercising at home followed instructions in a booklet. Everyone was provided with the appropriate equipment to enable them to perform the exercises.

### 2.5. Outcome Measures

Feasibility was determined by retention and adherence rates in both facility-based and home-based PEI [19]. The retention rate was calculated as the percentage of participants who completed the follow-up assessment at 12 weeks relative to those who completed the baseline assessment. The adherence rate was defined as the percentage of completed PEI sessions, calculated by dividing the sum of participants’ average adherence by the number of participants who completed the PEI program. Adherence with group work-out sessions was assessed during the time that they participated. Adherence to home-based PEI was obtained by self-reporting based on exercise diaries. The PEI was deemed to be feasible if the following criteria were met: 1) a minimum retention rate of 75% at week 12 and 2) minimum adherence to the PEI of 75%.

The safety of the intervention was monitored during the supervised sessions and by inspection of the exercise diaries. Furthermore, cognitive function, mood, and physical performance were compared for each participant before and after the 12 week PEI. Cognitive function was evaluated using the K-MMSE, Alzheimer’s Disease Assessment Scale cognitive subscale (ADAS-Cog) [20], and the story memory test and stick construction test of the Literacy Independent Cognitive Assessment (LICA) [21]. Mood status was assessed using the Korean version of the short form of the Geriatric Depression Scale (SGDS-K) [22]. Physical performance was assessed by the physical fitness test battery for the elderly used in the Korean National Physical Performance Evaluation Program as well as the Short physical performance battery, which consists of three parts (standing balance, gait speed, and repeated chair stands) [23].

### 2.6. Statistical Analysis

The demographic and clinical characteristics of the participants were summarized as descriptive statistics. Paired *t*-test analysis was performed to assess changes in cognitive function and physical fitness after 12 weeks for both facility-based and home-based PEI. Cognitive/physical items that were confirmed by the paired *t*-test to have improved after PEI were entered into a multiple linear regression analysis to investigate whether there was any significant independent predictor among the variables, such as age (continuous variable), sex, a Clinical Dementia Rating (CDR) score of 0 or 0.5 [24], adherence (≥75% or <75%), baseline physical fitness (fair or poor), or physical activity (active or inactive).

## 3. Results

### 3.1. Demographic and Clinical Characteristics of Participants

Four of 30 individuals screened were excluded from participation in the study because of a need for a cane to ambulate (*n* = 1), recent medical history of pacemaker insertion due to arrhythmia (*n* = 1), recent rotator cuff tear injury (*n* = 1), and diagnosis of cancer that was being treated (*n* = 1). The remaining 26 individuals were assigned to facility-based PEI (*n* = 15) or home-based PEI (*n* = 11; Table 2). Table 2 shows the demographic and clinical characteristics of the participants. The mean age was 67.9 years and 84.6% of participants were female. The mean number of years of education was 11.6. The mean K-MMSE score was 27.3; 53.8% of participants scored 0 on the global CDR and the remainder scored 0.5. Eight individuals (30.7%; four each in the facility-based and home-based PEI programs) were assessed to be physically inactive by the GPAQ. Assessment of physical performance showed that seven individuals (26.9%; four in the facility-based group and three in the home-based group) had poor physical fitness. In the facility-based group, two participants were below the 30th percentile of the norm for flexibility and the other two participants for coordination. In the home-based group, three participants showed poor leg strength/endurance (*n* = 1), poor flexibility (*n* = 1), or poor flexibility and cardiopulmonary endurance (*n* = 1). Moreover, in the home-based group, a strength-intensive program was recommended for five individuals and an aerobic exercise-intensive program for six.

### 3.2. Feasibility of the 12 Week PEI

**Figure 2:**
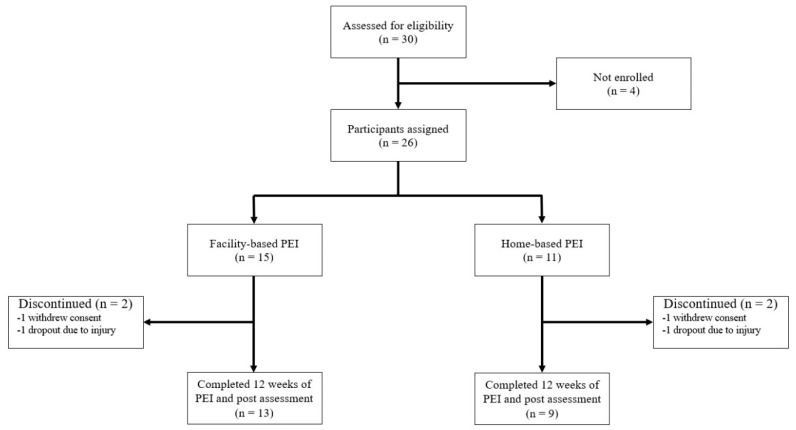
Trial profile. PEI, physical exercise intervention.

Four participants (two from each group) dropped out for personal reasons (*n* = 2) or because of injuries not related to the PEI. Therefore, 13 participants (86.7%) in the facility-based group and 9 (81.8%) in the home-based group completed the 12 week PEI program (Figure 2).

The 12-week adherence rate was 88.3% for the facility-based PEI and 62.3% for the home-based PEI. Table 3 summarizes the adherence rate data calculated at 3 week intervals. In the facility-based group, the adherence rate remained steady at above 80% with an 8% drop between the first and final 3 week periods. In the home-based group, the adherence rate was 32.1% during the first 3 weeks but increased thereafter, reaching 81.5% in the final 3 weeks.

No safety issues related to the PEI program occurred during the 12-week study period.

### 3.3. Changes in Cognitive Function and Physical Fitness after the 12 Week PEI

At the end of the study, there was a significant improvement in leg strength/endurance and cardiopulmonary endurance in both groups (Table 4), as evidenced by the results of the 30 s sit-to-stand test (facility-based PEI, 20.6 ± 3.0 vs. 23.7 ± 3.7, *p* = 0.002; home-based PEI, 18.9 ± 4.3 vs. 23.2 ± 3.9, *p* = 0.002) and 2 min stationary march (facility-based PEI, 109.3 ± 13.8 vs. 120.8 ± 15.7, *p* = 0.001; home-based PEI, 104.7 ± 14.5 vs. 120.3 ± 19.0, *p* = 0.022). The results for the 3 m sit–walk-and-return test indicated improvement in balance in the facility-based group. However, cognitive function improved only in the facility-based group (ADAS-Cog total score, 11.6 ± 4.2 vs. 9.2 ± 3.7, *p* = 0.009; story memory test in the LICA, 12.0 ± 5.1 vs. 14.8 ± 2.6, *p* = 0.026; Table 4).

Multiple linear regression revealed that only global CDR was an independent predictor of changes in the ADAS-Cog total score in the facility-based group (B = 4.337, SE = 1.630, *p* = 0.038). The ADAS-Cog total score improved more in the CDR 0.5 group (14.0 ± 2.8 vs. 11.5 ± 2.3) than in the CDR 0 group (9.6 ± 3.8 vs. 7.3 ± 3.7) after PEI. No other significant independent predictor was identified.

## 4. Discussion

Our PEI is a multicomponent and structured physical exercise program developed by medical and sports science professionals. The facility-based PEI was demonstrated to be safe and feasible with retention and adherence rates of over 75% and was able to prevent dementia by improving physical fitness and cognitive function in the at-risk elderly population. However, our home-based PEI program needs some complementary measures to ensure that participants are motivated and do not feel that it is difficult to carry out the PEI program on their own, to increase its adherence rate.

This success of our facility-based PEI program may lie in the fact that it is tailored to the cultural and physical characteristics of the elderly population in Korea. It can be performed in public facilities with portable aids and includes various modes of exercise, including aerobic, resistance, balance, or stretching exercises, rather than focusing only on aerobic exercise. Our retention and adherence rates for facility-based PEI were 86.7% and 88.3%, respectively. The adherence rate in the final three weeks was 84.6%. Our finding of high retention and adherence rates during 12 weeks of facility-based PEI has feasibility implications, because it is known that sustaining exercise habits for as long as possible promotes cognitive brain health through interest and a sense of accomplishment [12]. A systematic review of studies of practical prescriptive exercise regimens revealed that the most important variable in terms of improved cognition was total exercise intervention time [12], irrespective of whether the exercise was aerobic, resistance (strength) training, mind–body exercises, or combinations of these interventions. Exercising for a total of at least 52 h is likely to improve cognitive performance in older adults, whether or not they have cognitive impairment; however, there was no relationship between cognitive improvement and session time, frequency, intensity, or weekly minutes. Therefore, a practical exercise regimen for cognitive benefit in the elderly should be tailored to the cultural and physical characteristics of the individual patient and include various modes of exercise and exercising for as long as possible. Although our facility-based PEI had good feasibility, measures to improve the withdrawal rate of 13.3% and decrease in adherence rate of 8% over 12 weeks could be necessary in longer trials. The inclusion of a motivation enhancement program in SUPERBRAIN seemed to be helpful for maintaining adherence to PEI [11].

Unlike facility-based PEI, there are some concerns regarding compliance with home-based PEI. Adherence with our home-based PEI program over 12 weeks was 62.3%, which is lower than the rate of 82.9% in another report [25]. However, by looking at the pattern of change in adherence over time in our study, we understood how to improve adherence with home-based PEI. For the first three weeks, the adherence rate was 32.1% but improved from week 4 onwards and reached 81.5% in the final three weeks. Early on in the program, our participants may have had trouble performing exercise at home with only booklets for guidance even though exercise professionals had provided tips on how to incorporate the exercises at home. Therefore, in SUPERBRAIN, in addition to the motivation enhancement program, tablets with exercise movies performed in group workouts were distributed [11].

After PEI, improvement in several indicators of efficacy, particularly leg strength/endurance and cardiopulmonary endurance, were also observed. Furthermore, the ADAS-Cog total score, an index of cognitive function, improved in participants who undertook facility-based PEI, and improved significantly more in the CDR 0.5 group than in the CDR 0 group. This suggests that the improvement in ADAS-Cog total score after PEI is not a result of the learning effect on the evaluation method, but that of the PEI efficacy. Moreover, when compared with the findings of previous studies, such as the Multidomain Alzheimer Preventive Trial [26,27] or FINGER [2,28], our results suggest that lifestyle intervention to prevent cognitive decline could be more beneficial in individuals at higher risk for dementia.

Our study has some limitations. First, adherence with home-based PEI was assessed by self-reports only. Second, the sample size was small, which limits the generalizability of its findings. The results of this study suggest that, whereas facility-based PEI could be feasible, a home-based program would need to include strict monitoring or more helpful reference material for exercise at home to improve the adherence rate before it is implemented as part of SUPERBRAIN.

## Figures and Tables

**Table 1:** Components and progression of the exercise program.

	Weeks 0–4	Weeks 5–8	Weeks 9–12
Structured exercise program	Level 1	Level 2	Level 3
Exercise frequency, per week	3	3	3
Percentage of maximum heart rate	40–50%	45–55%	50–60%
Duration of exercise (minutes/session)	60	60	60
Resistance exercise (minutes/session)	20–25	20–25	20–25
Muscle groups, n	10	12	15
Sets, n	1–2	1–3	2–4
Aerobic exercise (minutes/session)	20–25	20–25	20–25
Balance exercise (minutes/session)	5	5	5
Finger-and-toe movements (minutes/session)	5	5	5
Stretching exercise (minutes/session)	5	5	5

**Table 2:** Components and progression of the exercise program.

Variable	Total (*n* = 26)	Facility-Based (*n* = 15)	Home-Based (*n* = 11)
Age, years	67.9 ± 3.6	67.3 ± 3.8	68.3 ± 3.2
Sex, female, *n* (%)	22 (84.6%)	14 (93.3%)	8 (72.7%)
Education, years	11.6 ± 4.0	10.5 ± 3.2	13.0 ± 4.7
Hypertension, *n* (%)	12 (46.2%)	4 (26.7%)	8 (72.7%)
Hypercholesterolemia, *n* (%)	6 (23.1%)	6 (40.0%)	0 (0.0%)
Diabetes mellitus, *n* (%)	4 (15.4%)	3 (20.0%)	1 (9.1%)
Musculoskeletal disease, *n* (%)	6 (23.1%)	1 (6.7%)	5 (45.5%)
Body mass index	23.7 ± 2.7	23.0 ± 2.7	24.5 ± 2.7
K-MMSE	27.3 ± 1.5	27.0 ± 1.5	27.8 ± 1.5
CDR			
0, *n* (%)	14 (53.8%)	8 (53.3%)	6 (54.5%)
0.5, *n* (%)	12 (46.2%)	7 (46.7%)	5 (45.5%)
GPAQ, MVPA (min/week)	366.5 ± 310.1	326.0 ± 246.8	421.8 ± 386.4

Continuous variables are shown as the mean and standard deviation. CDR, clinical dementia rating; GPAQ, Global Physical Activity Questionnaire; K-MMSE, Korean version of the Mini-Mental State Examination; MVPA, moderate-to-vigorous physical activity.

**Table 3:** Adherence rate (%) calculated at 3 week intervals.

	Weeks 1–3	Weeks 4–6	Weeks 7–9	Weeks 10–12	Total
Facility-based PEI	92.3	90.4	86.3	84.6	88.3
Home-based PEI	32.1	64.2	71.6	81.5	62.3

PEI, physical exercise intervention.

**Table 4:** Changes in cognitive function and physical fitness after PEI.

Variable	Facility-Based (*n* = 13)				Home-Based (*n* = 9)			
	Pre	Post	*t*	*p*-Value	Pre	Post	*t*	*p*-Value
Cognition								
K-MMSE	27.1 ± 1.6	26.5 ± 2.0	0.846	0.414	27.9 ± 1.6	27.7 ± 1.9	0.555	0.594
ADAS-Cog, total score	11.6 ± 4.2	9.2 ± 3.7	3.121	0.009	8.8 ± 4.2	8.1 ± 4.7	0.784	0.455
LICA, word delayed recall	7.8 ± 2.8	8.9 ± 0.9	−1.656	0.124	8.7 ± 1.4	9.0 ± 1.4	−1.000	0.347
LICA, story delayed recall	12.0 ± 5.1	14.8 ± 2.6	−2.531	0.026	12.5 ± 4.4	13.0 ± 4.8	−0.418	0.687
LICA, stick reconstruction	16.8 ± 1.7	15.3 ± 6.4	0.830	0.420	17.1 ± 0.9	14.1 ± 7.0	1.364	0.202
SGDS-K	1.0 ± 1.3	0.8 ± 1.3	0.485	0.636	0.9 ± 1.3	0.3 ± 0.7	1.474	0.179
Physical performance								
Body mass index	23.0 ± 2.7	23.6 ± 2.9	−3.150	0.008	24.1 ± 2.8	24.7 ± 3.3	−3.155	0.014
Grip test, left (kg )	24.4 ± 3.3	24.4 ± 3.6	−0.080	0.937	24.5 ± 7.1	24.6 ± 6.1	−0.029	0.978
Grip test, right (kg)	24.7 ± 3.5	24.3 ± 3.8	0.853	0.410	24.1 ± 6.3	25.1 ± 5.6	−1.737	0.121
Relative grip strength (%)	45.4 ± 5.4	43.9 ± 5.7	1.921	0.079	43.1 ± 11.2	43.2 ± 10.5	−0.104	0.920
30 s sit–stand test (times)	20.6 ± 3.0	23.7 ± 3.7	−3.945	0.002	18.9 ± 4.3	23.2 ± 3.9	−4.526	0.002
3 m sit–walk-and-return test (sec)	4.7 ± 0.7	4.9 ± 0.7	−2.386	0.034	4.8 ± 0.6	5.0 ± 0.6	−2.144	0.064
Sit-and-reach test (cm)	16.6 ± 7.4	18.2 ± 6.5	−1.501	0.159	10.5 ± 12.5	11.6 ± 9.7	−0.766	0.466
2 min stationary march (times)	109.3 ± 13.8	120.8 ± 15.7	−4.488	0.001	104.7 ± 14.5	120.3 ± 19.0	−2.841	0.022
* Figure-8-walks (sec)	24.7 ± 3.7	23.7 ± 3.5	1.864	0.087	24.8 ± 3.9	24.8 ± 3.5	−0.035	0.973

K-MMSE, Korean version *of the Mini-Mental State Examination; ADAS-Cog, Alzheimer’s Disease Assessment Scale cognitive subscale; LICA, Literacy Independent Cognitive Assessment; SGDS-K, Korean version of the short form of the Geriatric Depression Scale. * Figure-8-walks, one of the physical fitness items, is for testing coordination. Walking around two cones arranged to resemble the figure ‘8′ involved marking the 1.6 m mark from the side of the chair, and placing cones at 1.8 m distances to the left and right of the cones, marking their furthest sides. Participants were asked to circle around the cone to their right side to sit down, and then stand immediately after to circle around the cone on their left side to approach the chair to sit down [18].

**Figure A1:**
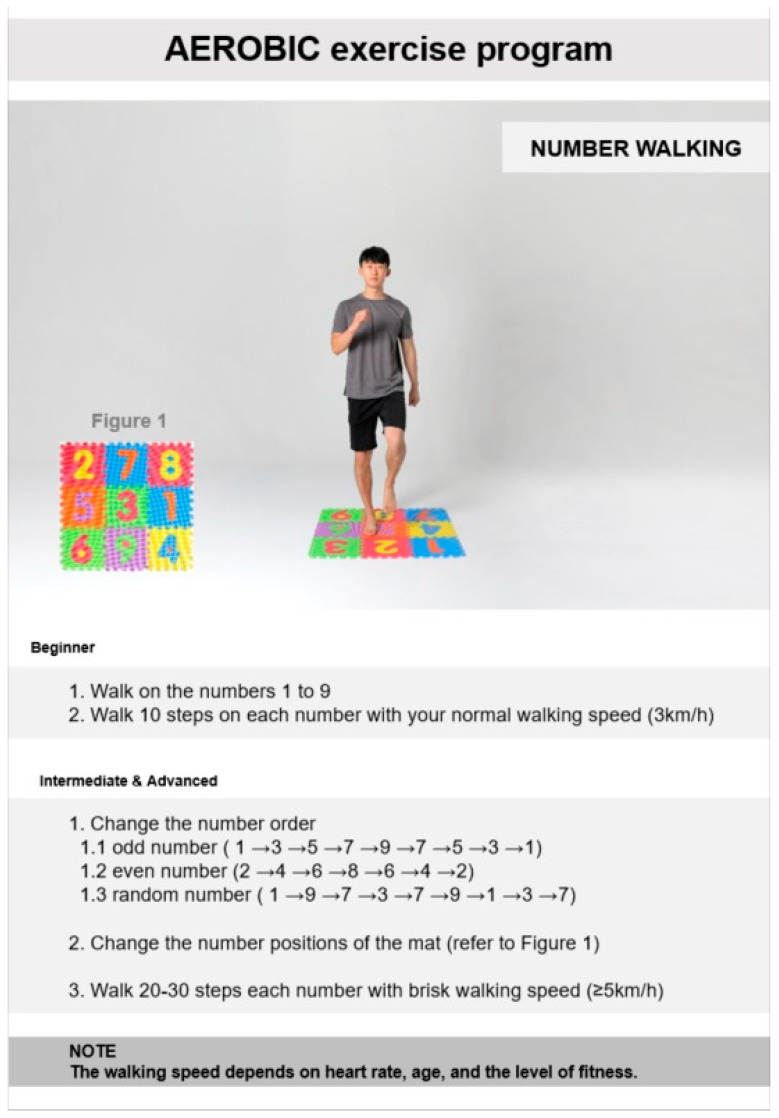
Detailed description and components of each movement in the “Number walking” aerobic exercise.

**Figure A2:**
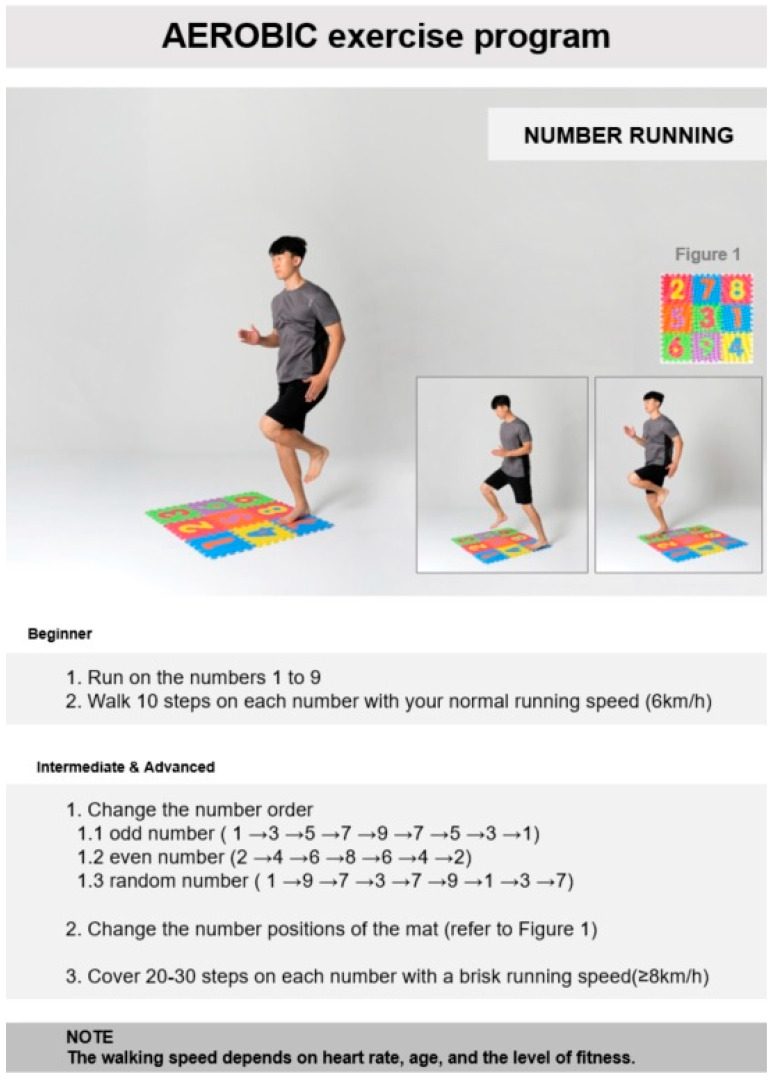
Detailed description and components of each movement in the “Number running” aerobic exercise.

**Figure A3:**
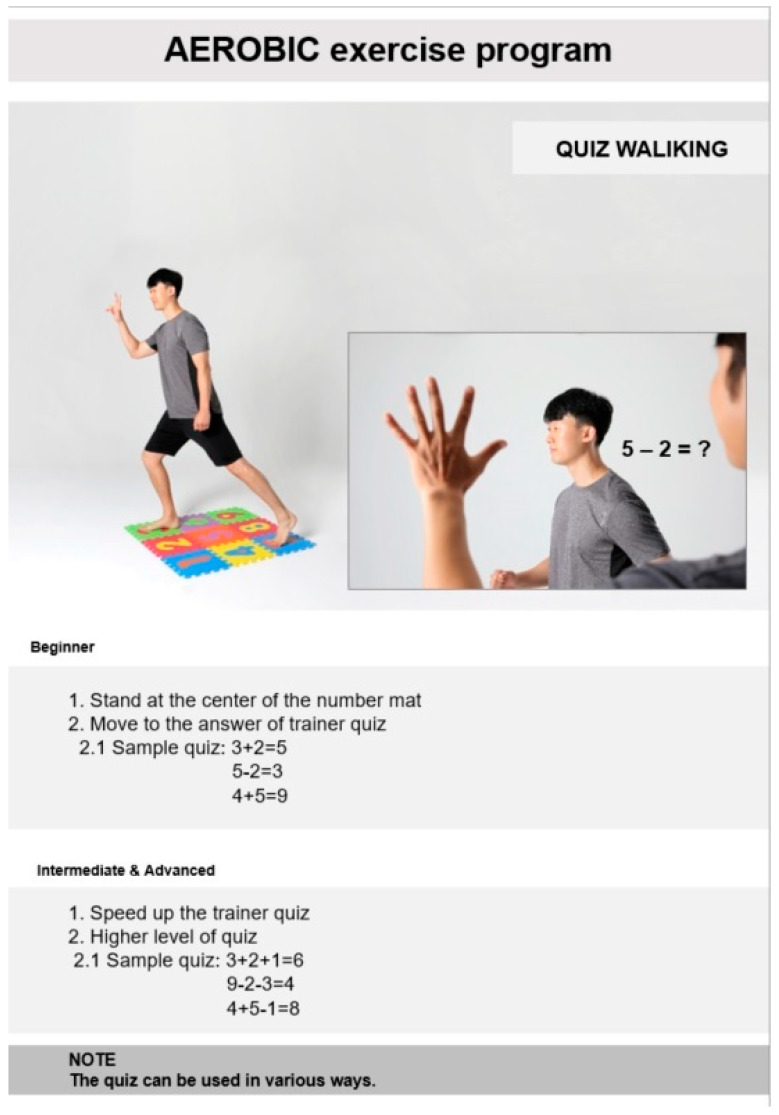
Detailed description and components of each movement in the “Quiz walking” aerobic exercise.

**Figure A4:**
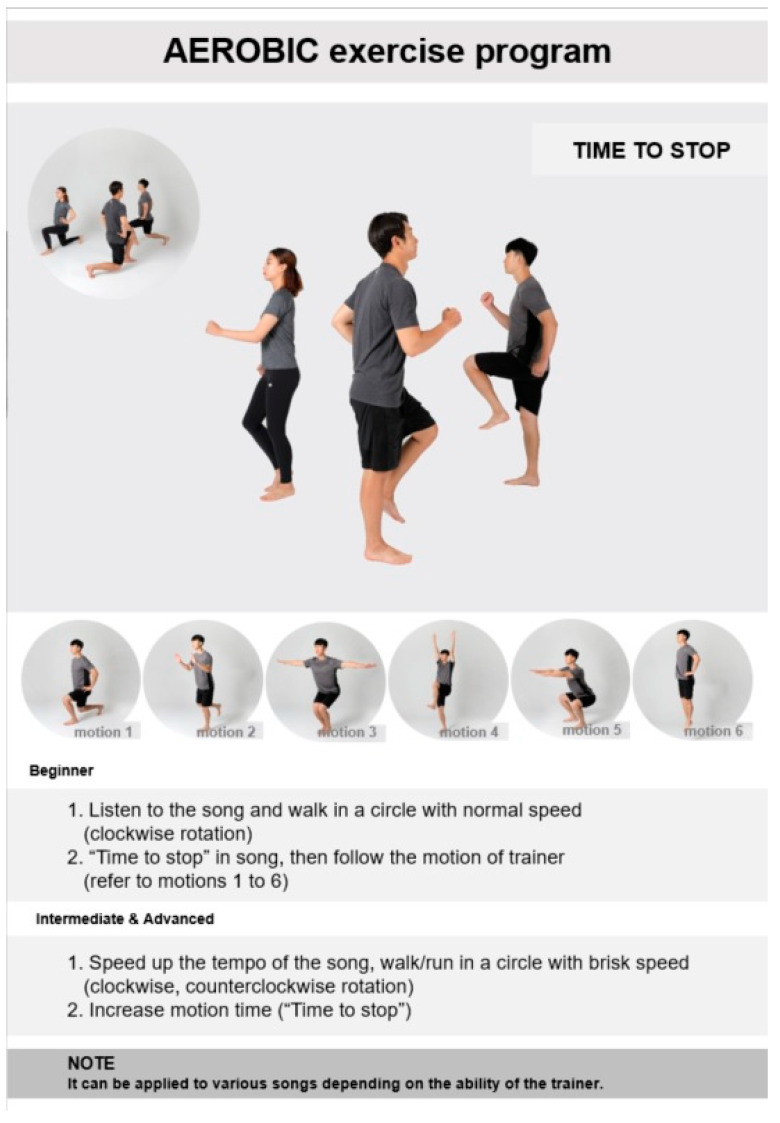
Detailed description and components of each movement in the “Time to stop” aerobic exercise.

**Figure A5:**
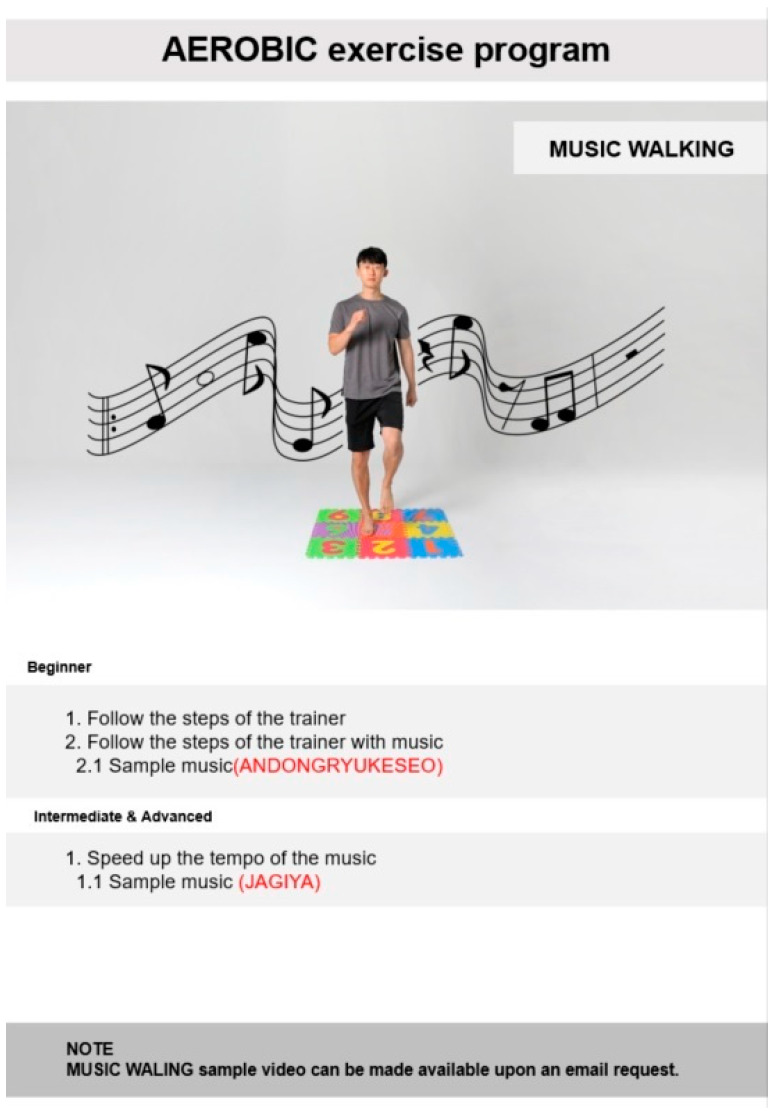
Detailed description and components of each movement in the “Music walking” aerobic exercise.

**Figure A6:**
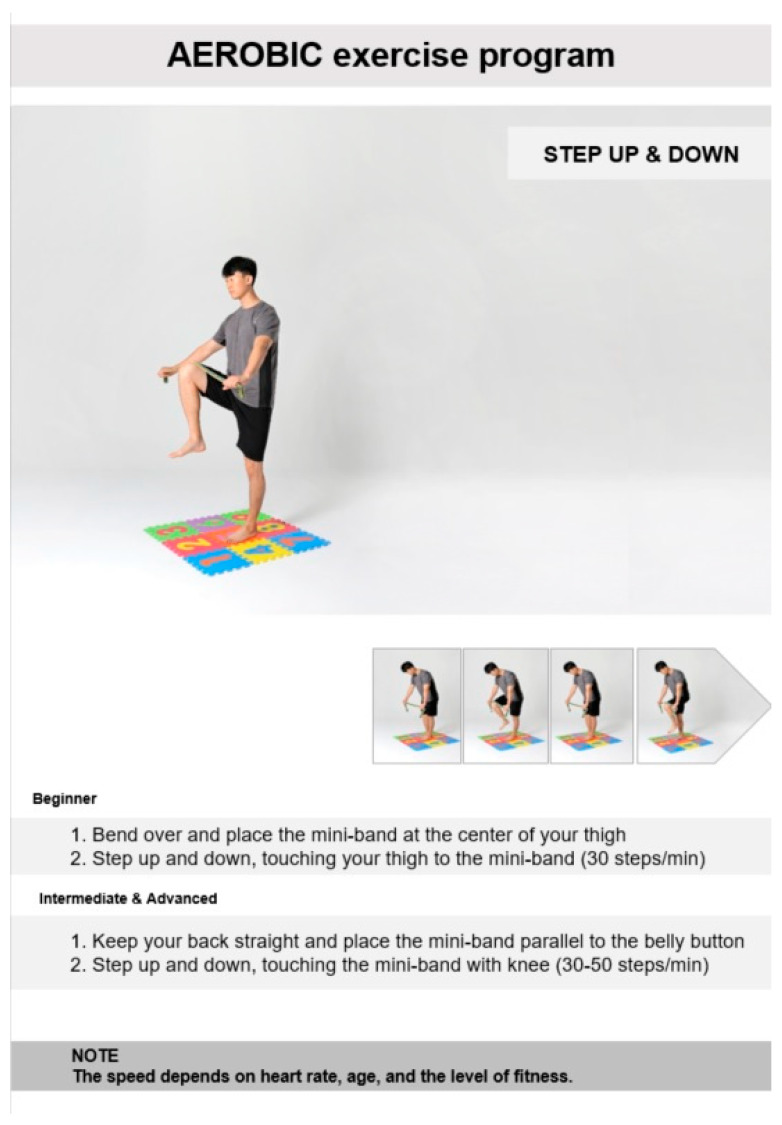
Detailed description and components of each movement in the “Step up and down” aerobic exercise.

**Figure A7:**
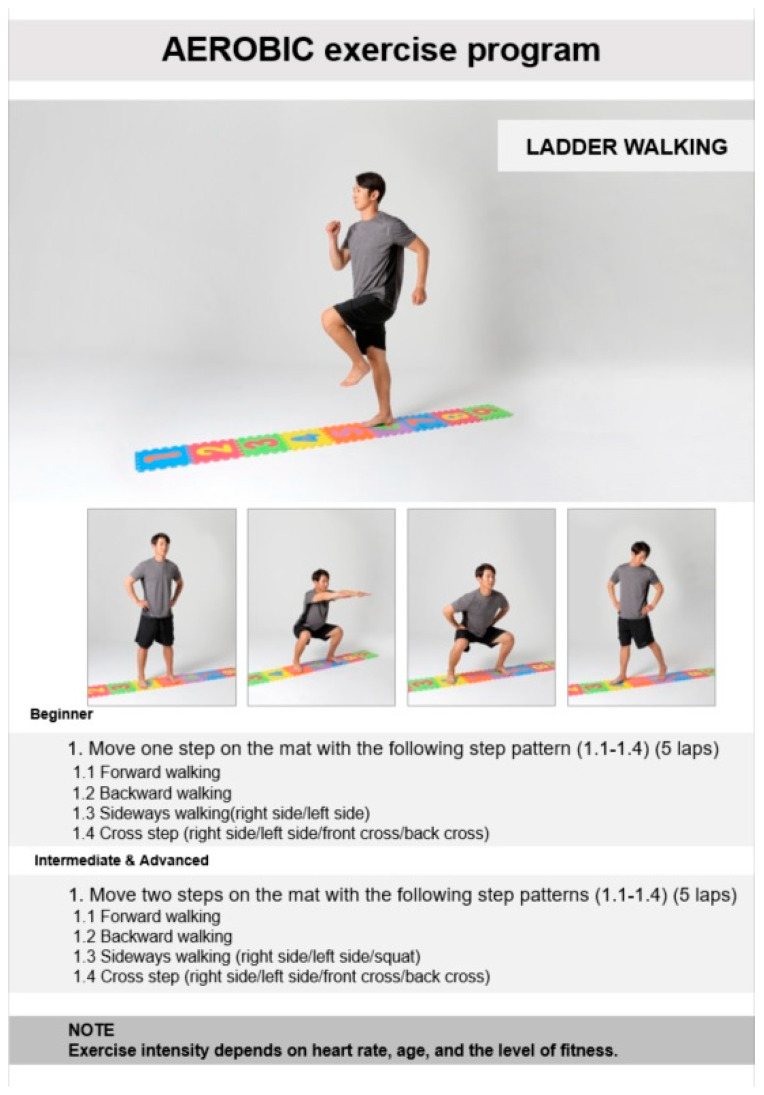
Detailed description and components of each movement in the “Ladder walking” aerobic exercise.

**Figure A8:**
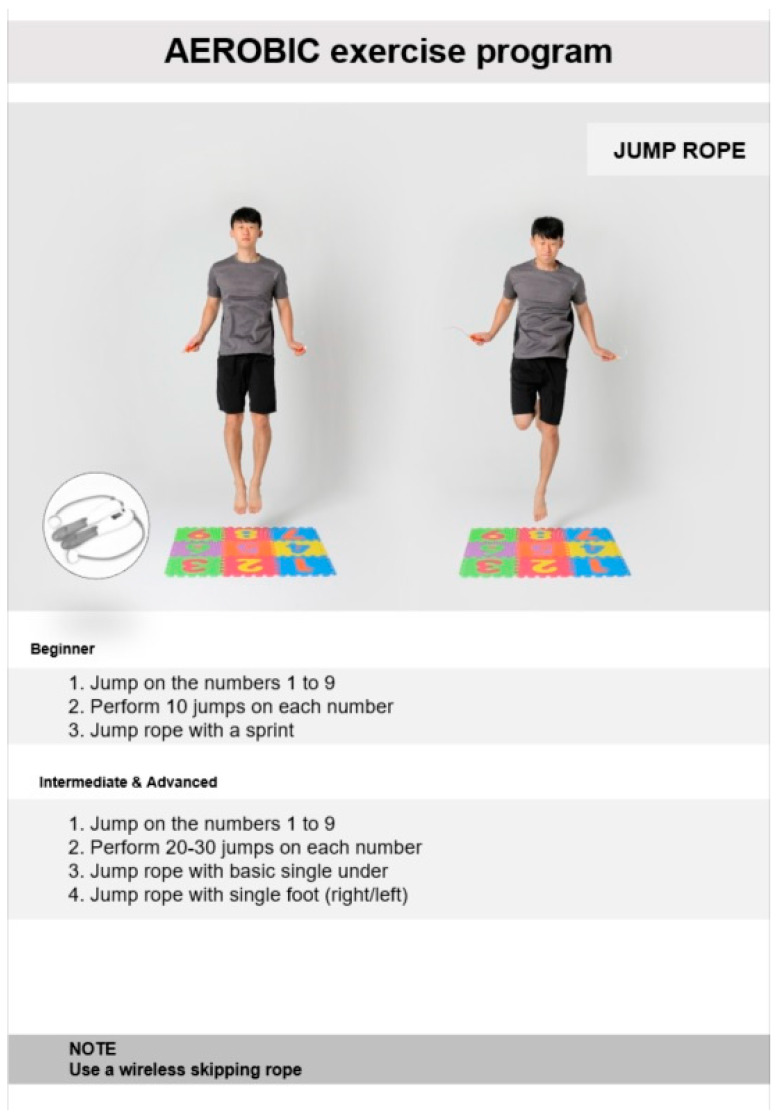
Detailed description and components of each movement in the “Jump rope” aerobic exercise.

**Figure A9:**
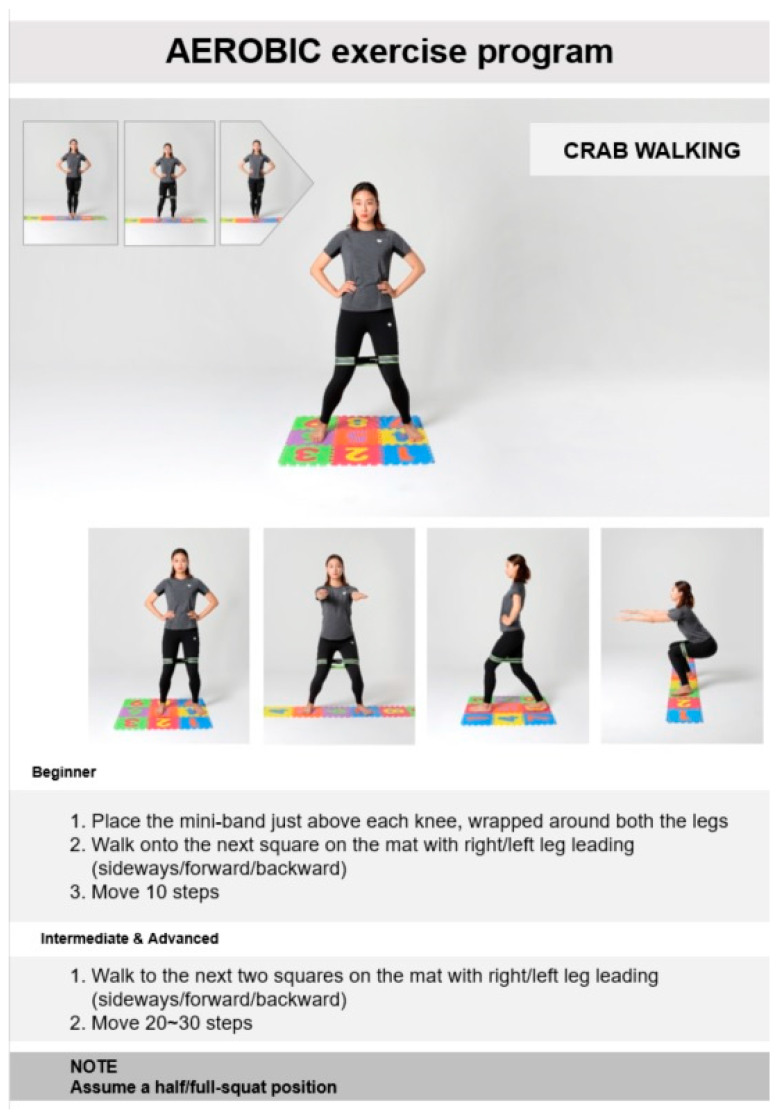
Detailed description and components of each movement in the “Crab walking” aerobic exercise.

**Figure A10:**
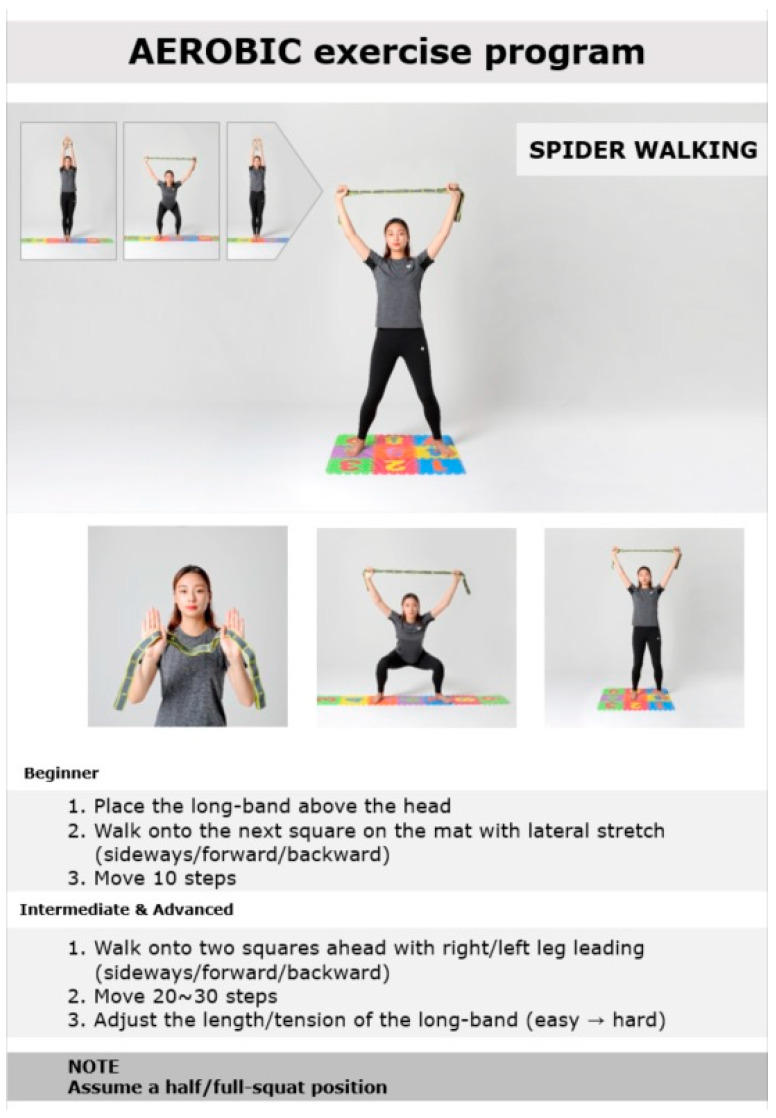
Detailed description and components of each movement in the “Spider walking” aerobic exercise.

**Figure A11:**
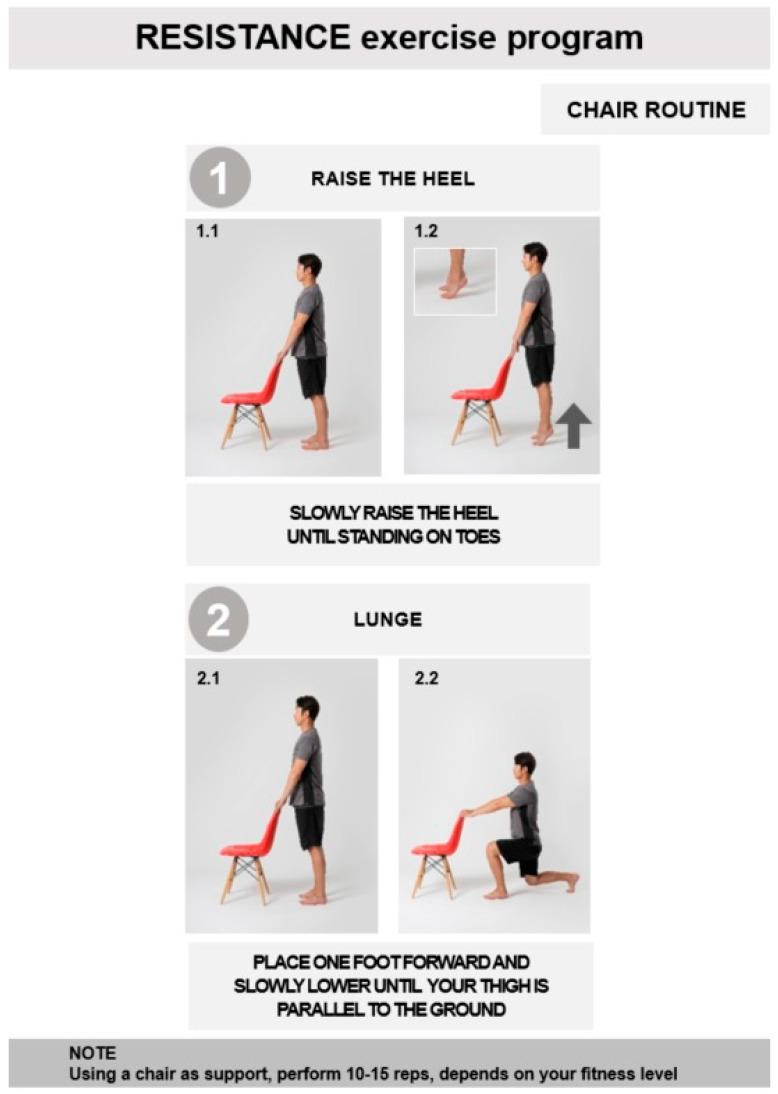
Detailed description and components of each movement in the “Chair routine part 1” resistance exercise.

**Figure A12:**
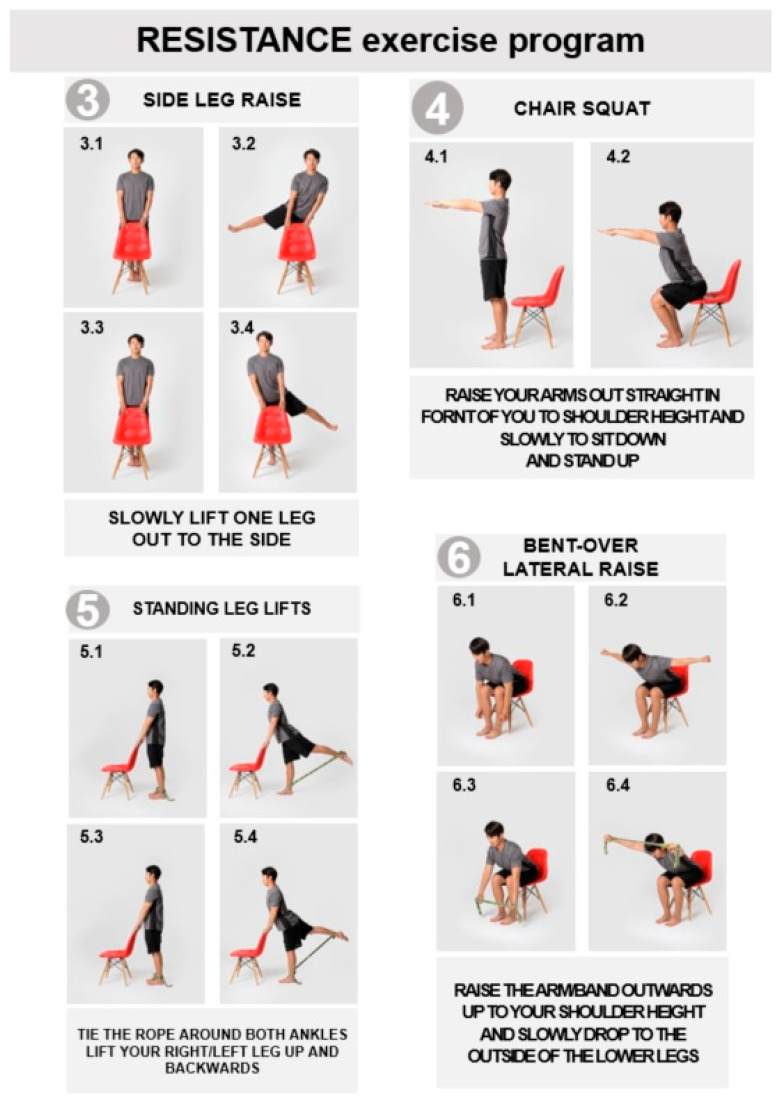
Detailed description and components of each movement in the “Chair routine part 2” resistance exercise.

**Figure A13:**
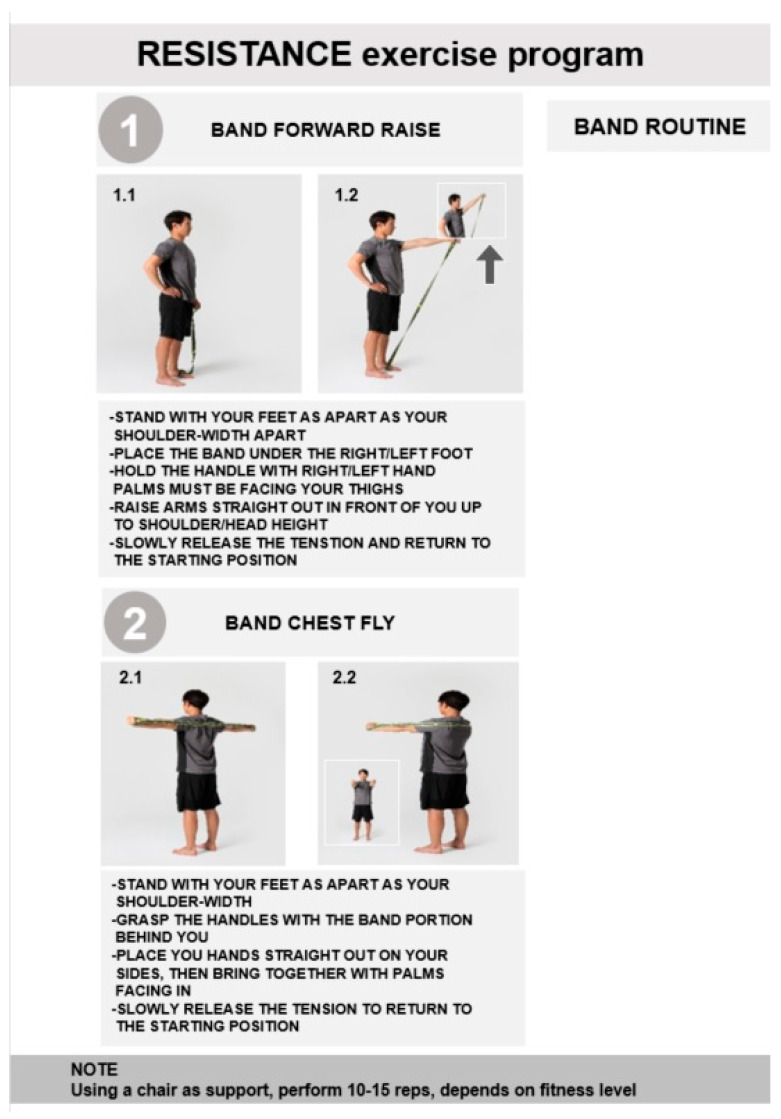
Detailed description and components of each movement in the “Band routine part 1” resistance exercise.

**Figure A14:**
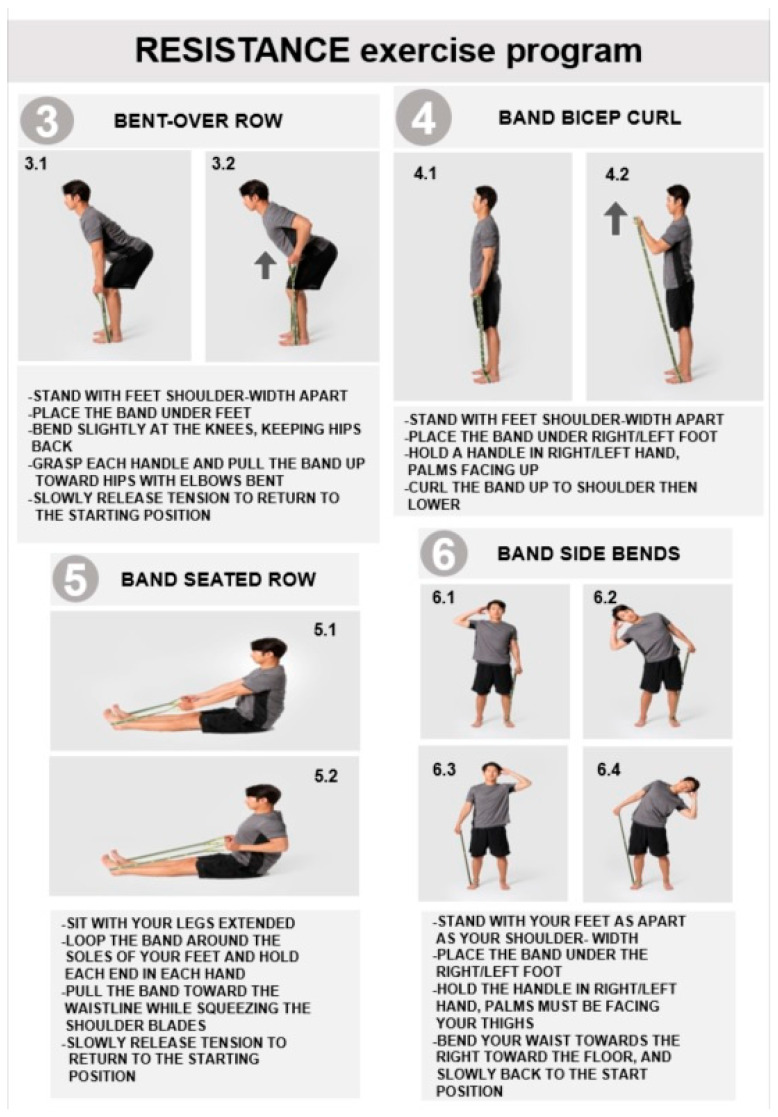
Detailed description and components of each movement in the “Band routine part 2” resistance exercise.

**Figure A15:**
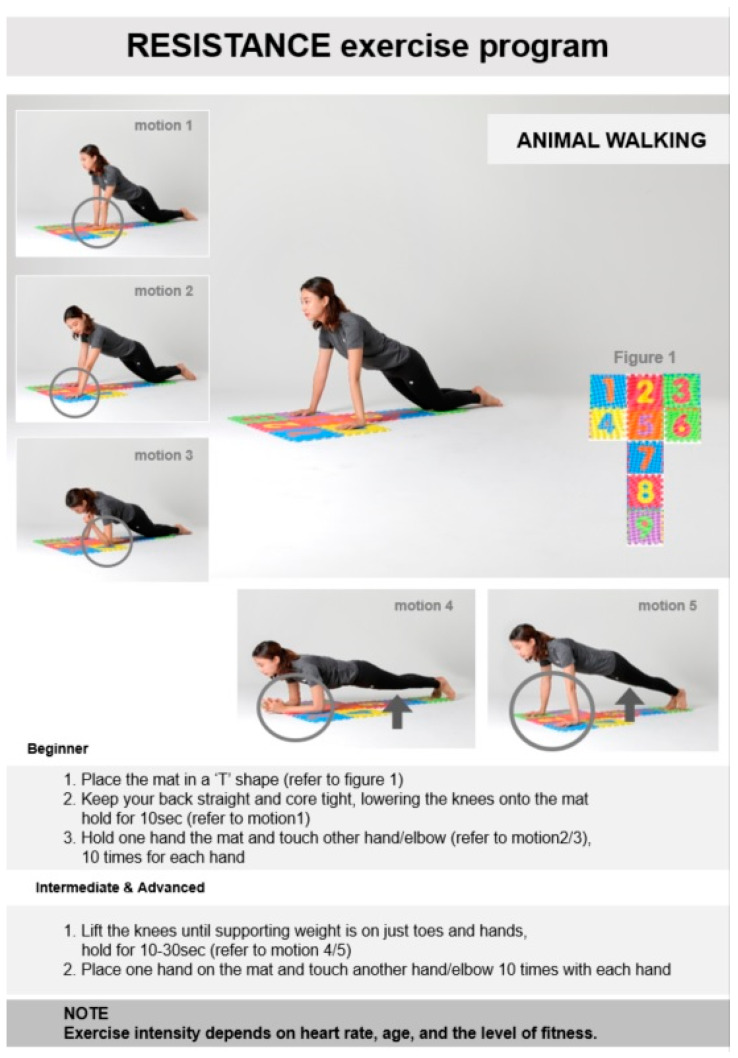
Detailed description and components of each movement in the “Animal walking” resistance exercise.

**Figure A16:**
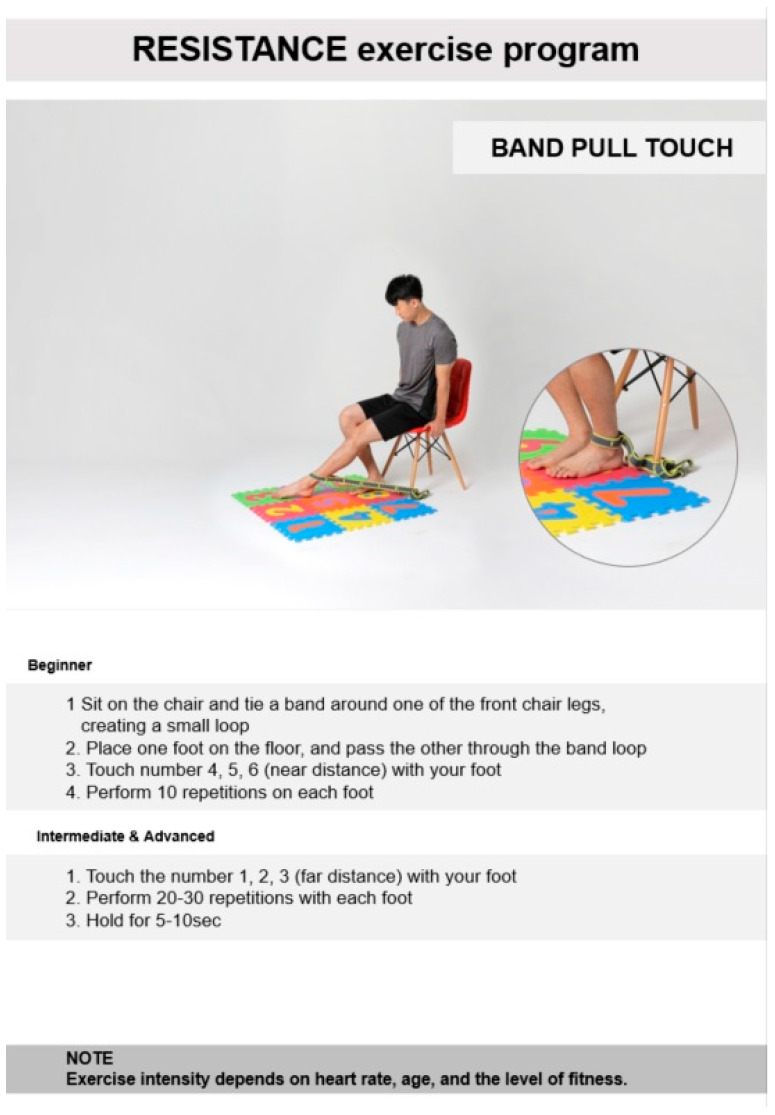
Detailed description and components of each movement in the “Band pull touch” resistance exercise.

**Figure A17:**
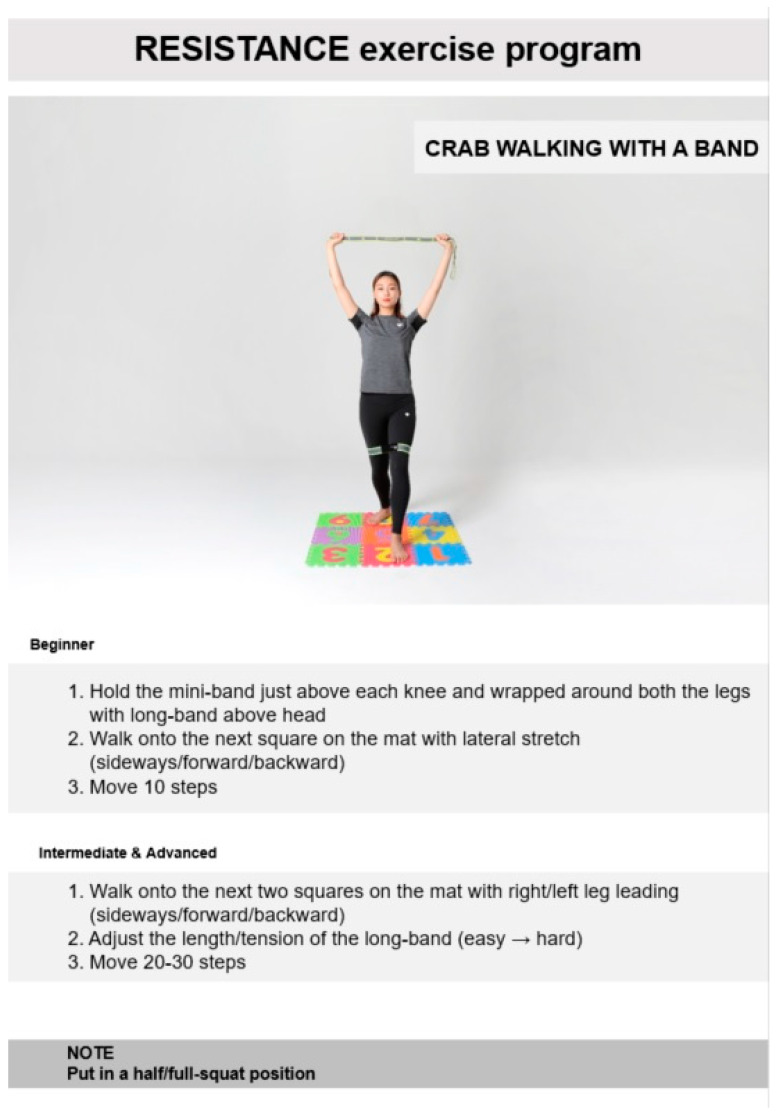
Detailed description and components of each movement in the “Crab walking with band” resistance exercise.

**Figure A18:**
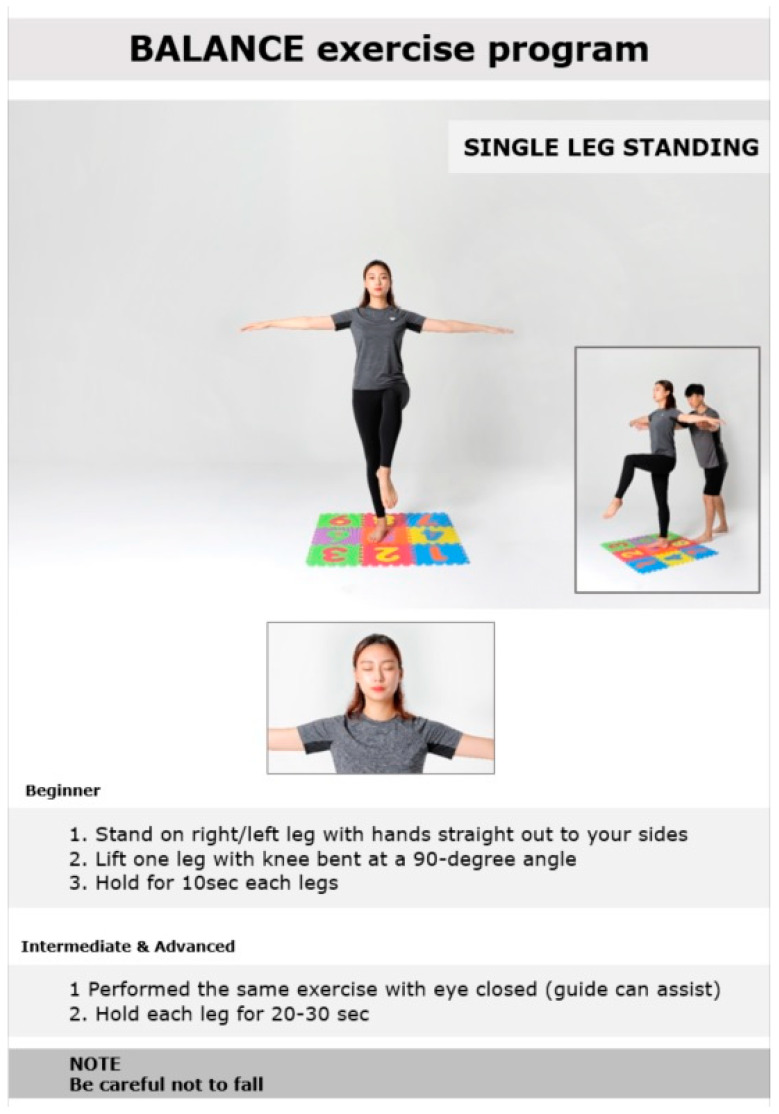
Detailed description and components of each movement in the “Single leg standing” resistance exercise.

**Figure A19:**
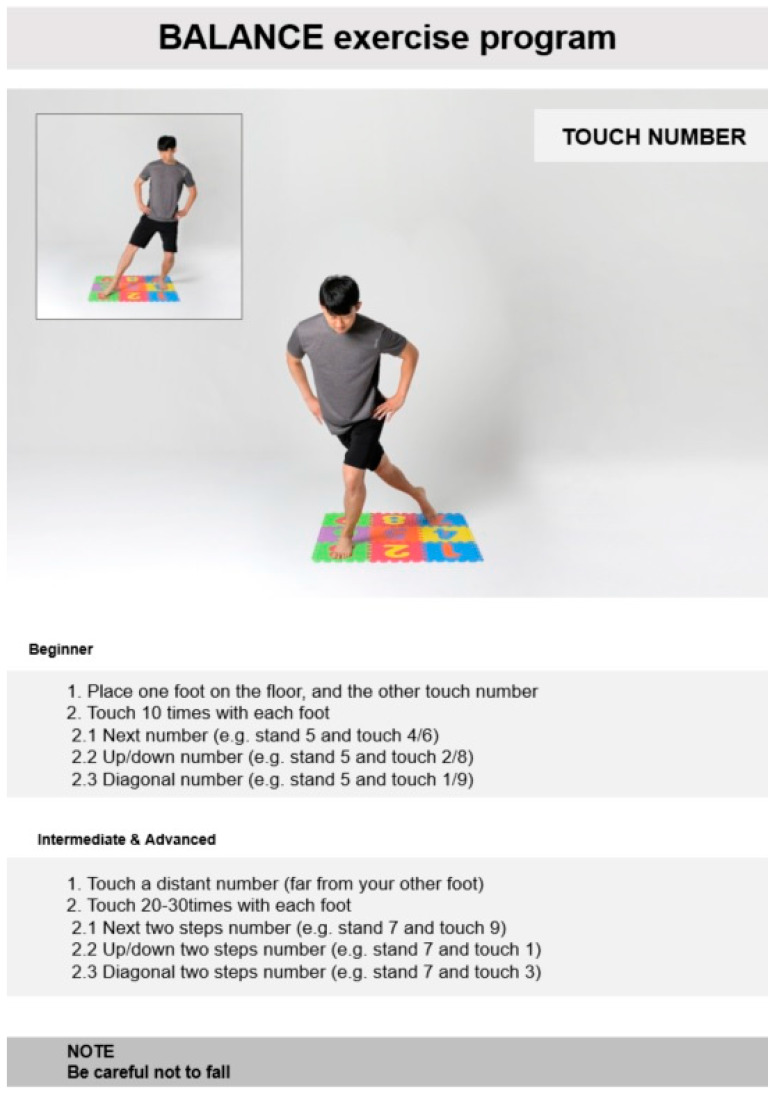
Detailed description and components of each movement in the “Touch number” balance exercise.

**Figure A20:**
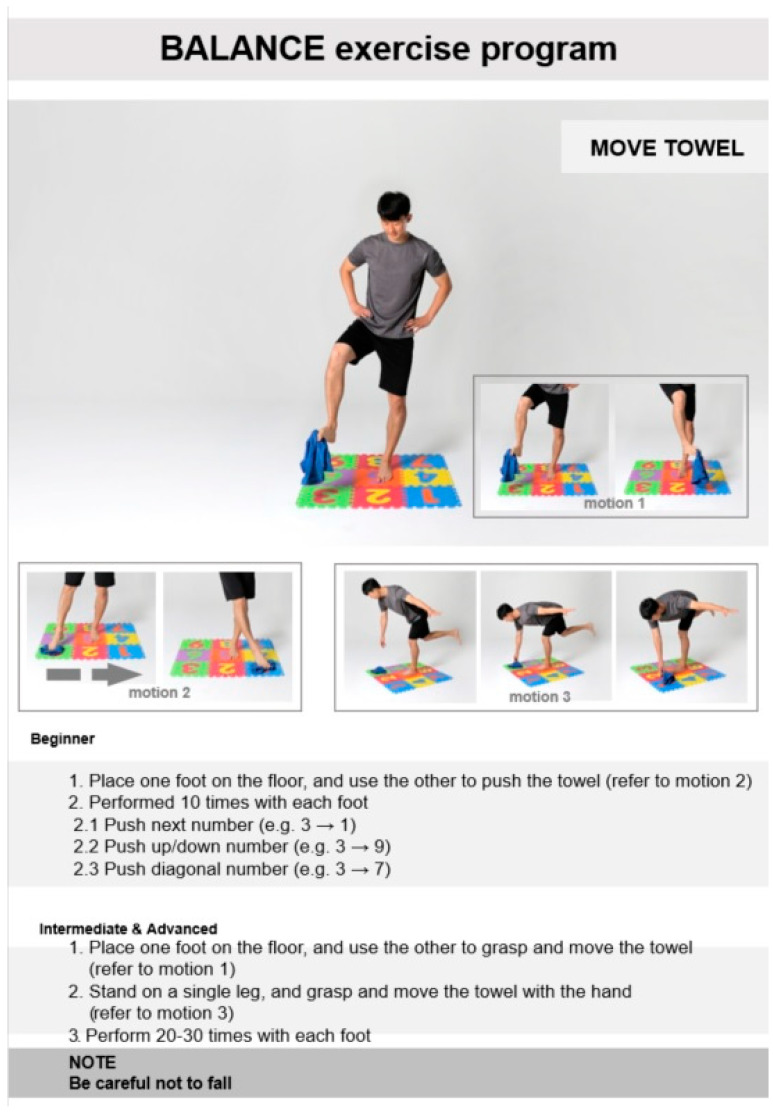
Detailed description and components of each movement in the “Move towel” balance exercise.

**Figure A21:**
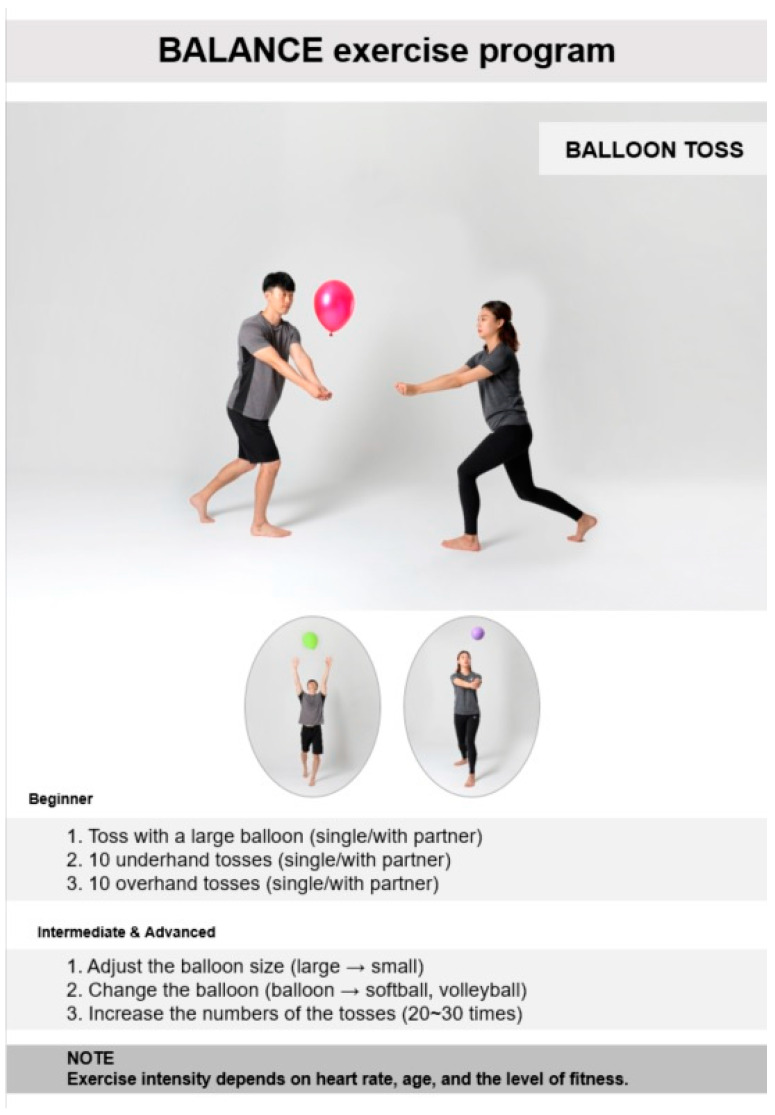
Detailed description and components of each movement in the “Balloon toss” balance exercise.

**Figure A22:**
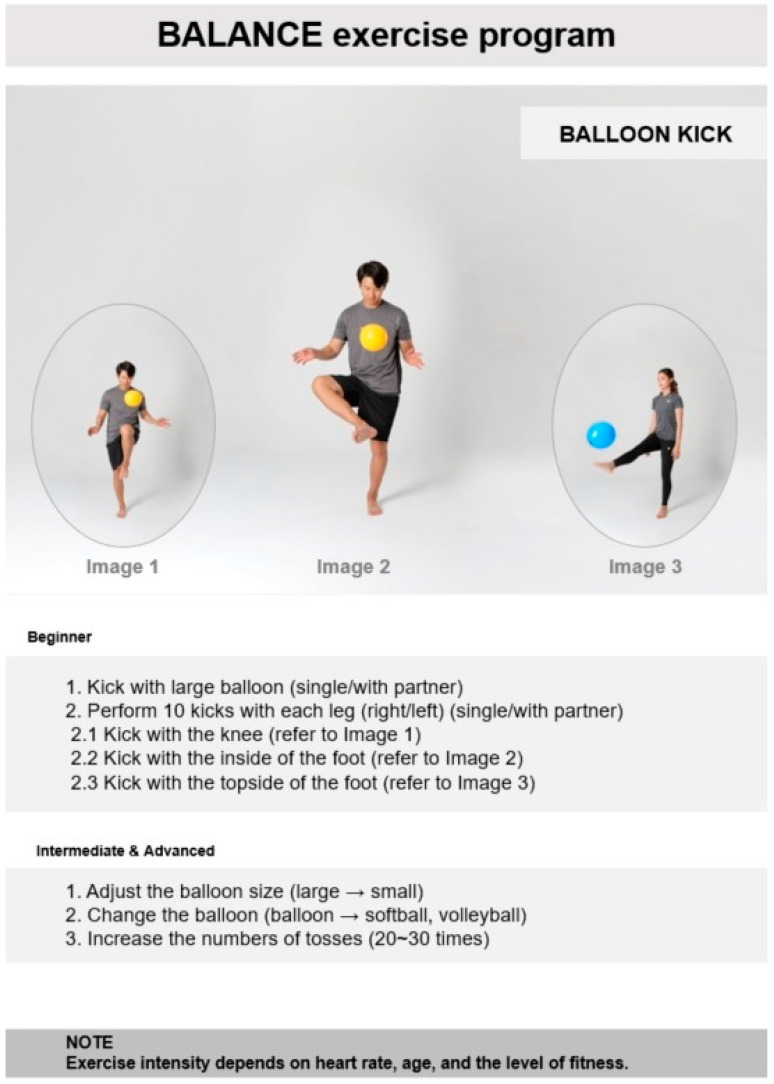
Detailed description and components of each movement in the “Balloon kick” balance exercise.

**Figure A23:**
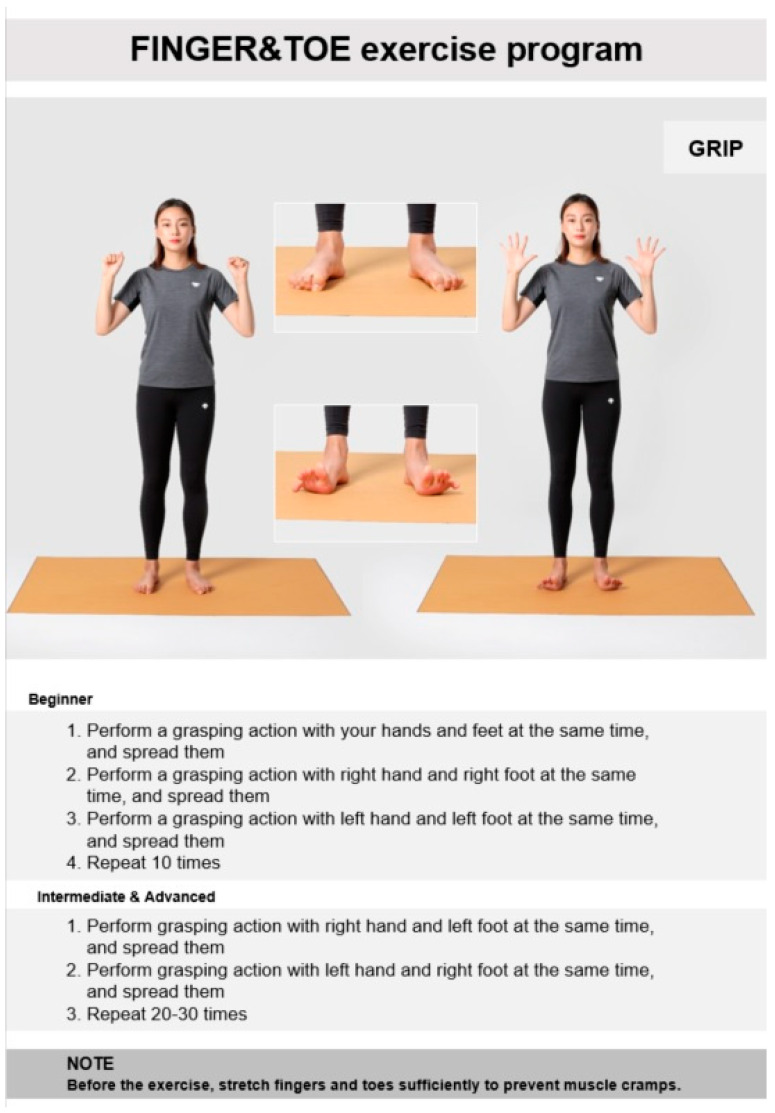
Detailed description and components of each movement in the “Grip” finger-and-toe exercise.

**Figure A24:**
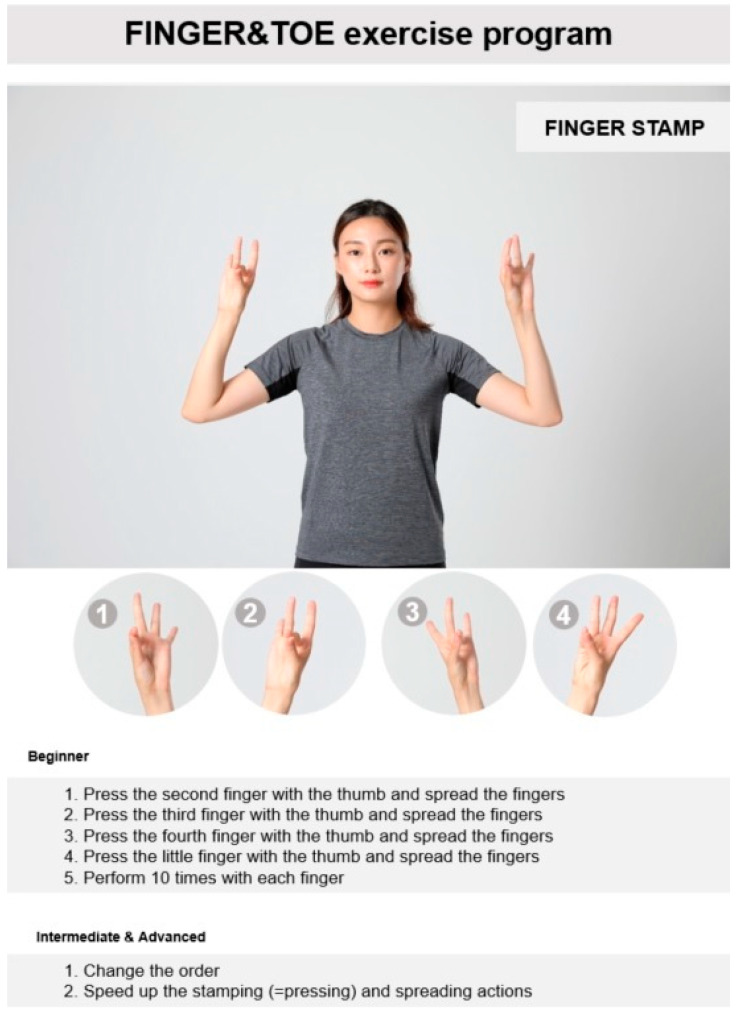
Detailed description and components of each movement in the “Finger stamp” finger-and-toe exercise.

**Figure A25:**
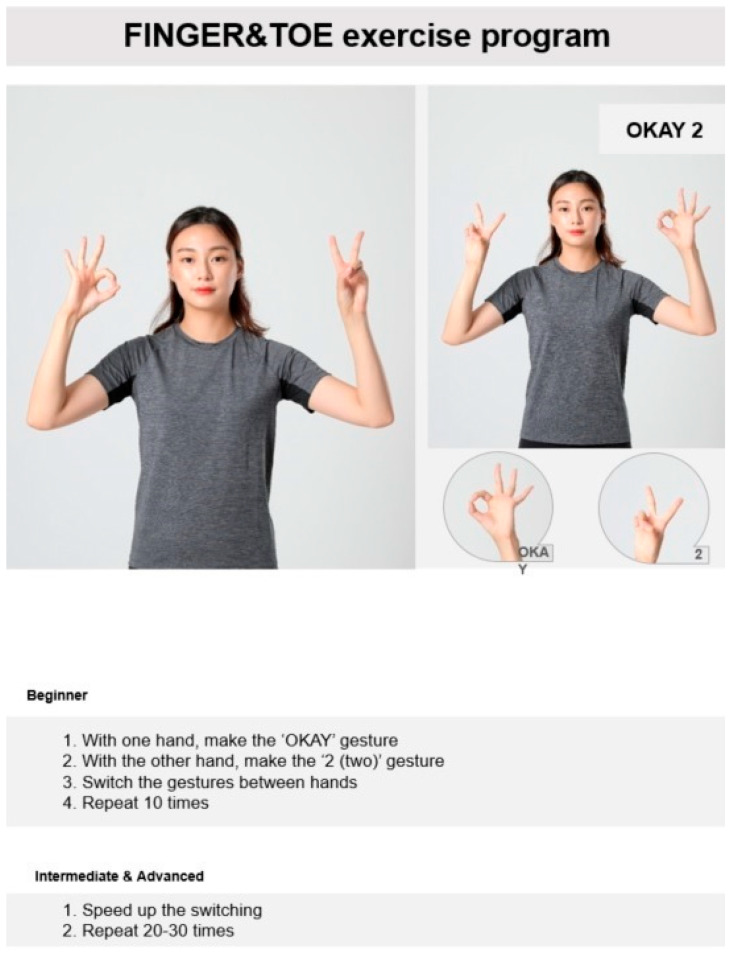
Detailed description and components of each movement in the “Okay 2” finger-and-toe exercise.

**Figure A26:**
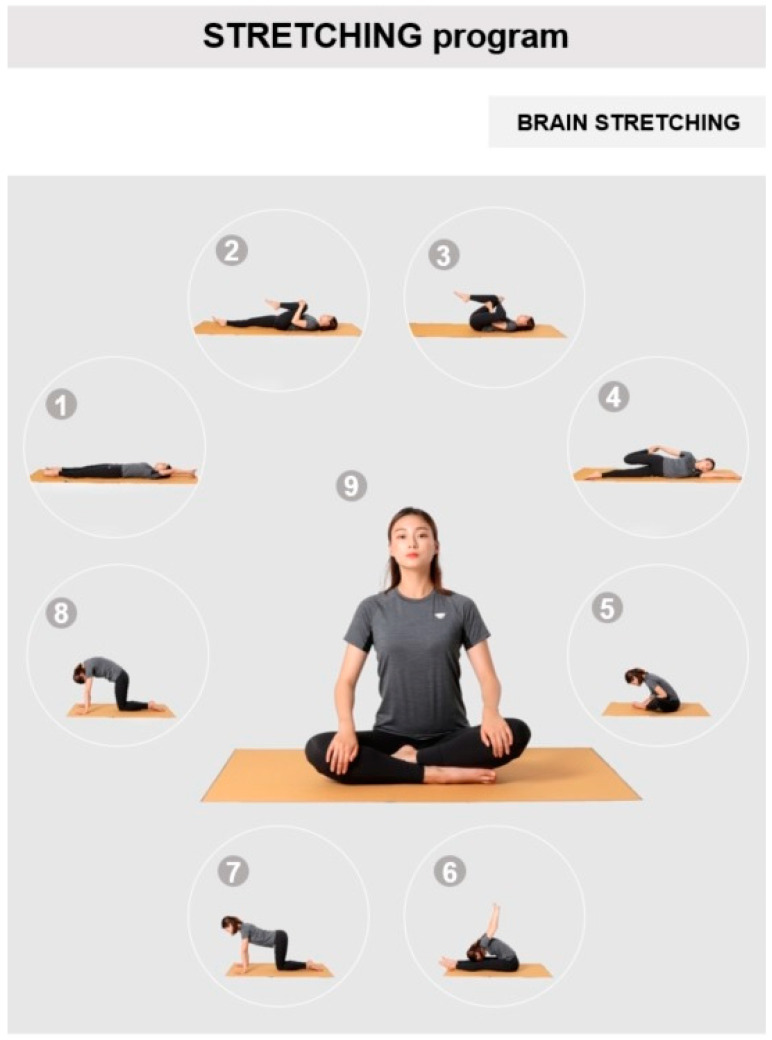
Detailed description and components of each movement in the “Brain stretching” stretch exercise.

**Figure A27:**
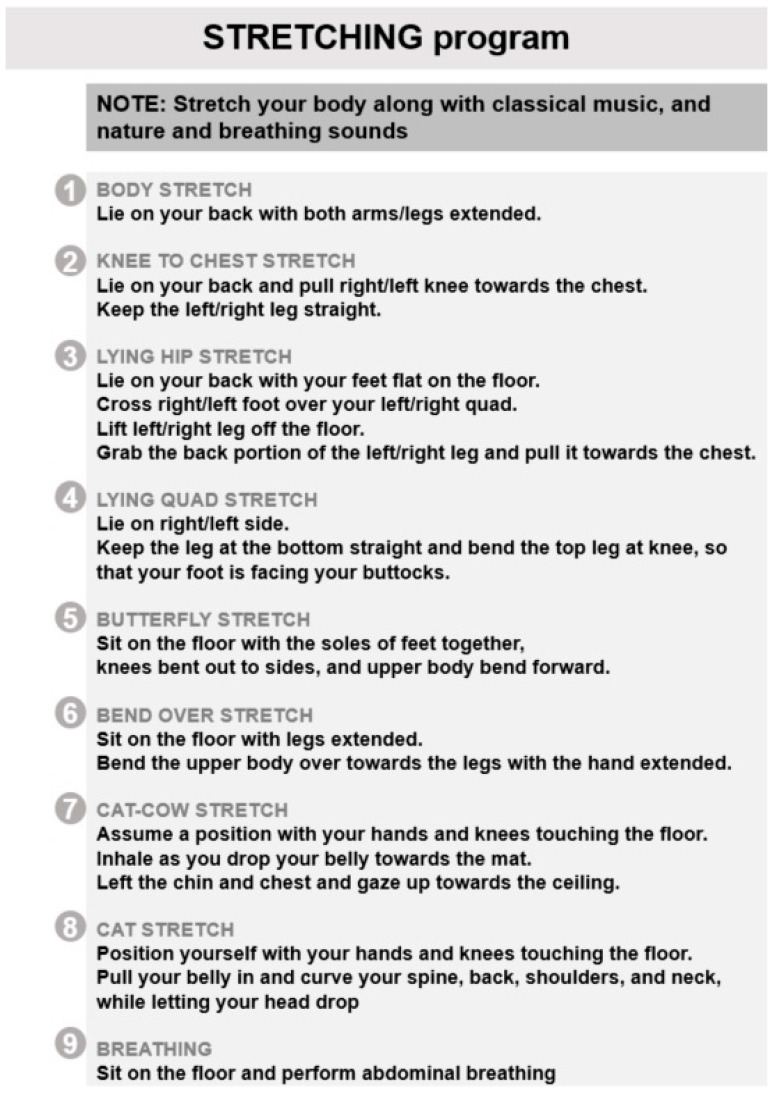
Detailed description of movements performed in the stretching program.

## Appendix A

**Table A1:** Detailed description of exercises.

	Group Session			Home Session					
				Strength-Intensive			Aerobic Exercise-Intensive		
Exercise category/name	L1	L2	L3	L1	L2	L3	L1	L2	L3
Aerobic exercise									
Number walking	●	●		●	●	●	●	●	●
Number running	●	●		●	●	●	●	●	●
Time to stop	●			●			●		
Quiz walking		●		●			●		
Crab walking						●		●	
Spider walking		●				●	●		
Jump rope			●		●	●	●	●	●
Ladder walking			●						
Step up and down	●	●	●	●	●	●	●	●	●
Music walking			●			●			●
Resistance exercise									
Band routine	●	●	●	●	●	●	●		●
Chair routine	●	●	●	●	●	●		●	
Animal walking				●			●		●
Band pull touch					●				
Crab walking with a band		●						●	●
Balance exercise									
Single leg standing	●		●		●				
Touch number	●		●						
Move towel	●		●	●					
Balloon toss		●			●			●	
Balloon kick		●			●			●	
Finger-and-toe exercise									
Grip	●				●		●	●	
Finger stamp		●			●	●	●	●	●
Okay 2			●			●			●
Stretching exercise									
Brain stretching	●	●	●	●	●	●	●	●	●

## References

[1] Kim Y.J., Han J.W., So Y.S., Seo J.Y., Kim K.Y., Kim K.W. (2014). Prevalence and trends of dementia in korea: A systematic review and meta-analysis. J. Korean Med. Sci..

[2] Chhetri J.K., de Souto Barreto P., Cantet C., Pothier K., Cesari M., Andrieu S., Coley N., Vellas B. (2018). Effects of a 3-year multi-domain intervention with or without omega-3 supplementation on cognitive functions in older subjects with increased caide dementia scores. J. Alzheimers Dis..

[3] Langa K.M., Larson E.B., Crimmins E.M., Faul J.D., Levine D.A., Kabeto M.U., Weir D.R. (2017). A comparison of the prevalence of dementia in the united states in 2000 and 2012. JAMA Intern. Med..

[4] Larson E.B., Yaffe K., Langa K.M. (2013). New insights into the dementia epidemic. N. Engl. J. Med..

[5] Lee Y., Back J.H., Kim J., Kim S.H., Na D.L., Cheong H.K., Hong C.H., Kim Y.G. (2010). Systematic review of health behavioral risks and cognitive health in older adults. Int. Psychogeriatr..

[6] Matthews F.E., Arthur A., Barnes L.E., Bond J., Jagger C., Robinson L., Brayne C. (2013). Medical Research Council Cognitive Function; Ageing Collaboration. A two-decade comparison of prevalence of dementia in individuals aged 65 years and older from three geographical areas of england: Results of the cognitive function and ageing study i and ii. Lancet.

[7] Ngandu T., Lehtisalo J., Solomon A., Levalahti E., Ahtiluoto S., Antikainen R., Backman L., Hanninen T., Jula A., Laatikainen T. (2015). A 2 year multidomain intervention of diet, exercise, cognitive training, and vascular risk monitoring versus control to prevent cognitive decline in at-risk elderly people (finger): A randomised controlled trial. Lancet.

[8] Norton S., Matthews F.E., Barnes D.E., Yaffe K., Brayne C. (2014). Potential for primary prevention of alzheimer’s disease: An analysis of population-based data. Lancet Neurol..

[9] Satizabal C.L., Beiser A.S., Chouraki V., Chene G., Dufouil C., Seshadri S. (2016). Incidence of dementia over three decades in the framingham heart study. N. Engl. J. Med..

[10] Schrijvers E.M., Verhaaren B.F., Koudstaal P.J., Hofman A., Ikram M.A., Breteler M.M. (2012). Is dementia incidence declining? Trends in dementia incidence since 1990 in the rotterdam study. Neurology.

[11] Park H.K., Jeong J.H., Moon S.Y., Park Y.K., Hong C.H., Na H.R., Song H.S., Lee S.M., Choi M., Park K.W. (2020). South korean study to prevent cognitive impairment and protect brain health through lifestyle intervention in at-risk elderly people: Protocol of a multicenter, randomized controlled feasibility trial. J. Clin. Neurol..

[12] Gomes-Osman J., Cabral D.F., Morris T.P., McInerney K., Cahalin L.P., Rundek T., Oliveira A., Pascual-Leone A. (2018). Exercise for cognitive brain health in aging: A systematic review for an evaluation of dose. Neurol. Clin. Pract..

[13] Kim J.H., Go S.M., Seo S.W., Kim S.H., Chin J., Moon S.Y., Lim H., Cheong H.K., Choi S.A., Lee J.H. (2015). Survival in subcortical vascular dementia: Predictors and comparison to probable alzheimer’s disease in a tertiary memory clinic population. Dement. Geriatr Cogn. Disord..

[14] Han C., Jo S.A., Jo I., Kim E., Park M.H., Kang Y. (2008). An adaptation of the korean mini-mental state examination (k-mmse) in elderly koreans: Demographic influence and population-based norms (the age study). Arch. Gerontol. Geriatr..

[15] Bull F.C., Maslin T.S., Armstrong T. (2009). Global physical activity questionnaire (gpaq): Nine country reliability and validity study. J. Phys. Act. Health.

[16] WHO (2011). Global Recommendations on Physical Activity for Health 18–64 Years Old.

[17] Jeong G.W., Kim Y.J., Park S., Kim H., Kwon O. (2019). Associations of recommended food score and physical performance in korean elderly. BMC Public Health.

[18] Park H., Park W., Lee M., Ko N., Kim E., Ishikawa-Takata K., Park J. (2018). The association of locomotive and non-locomotive physical activity measured by an accelerometer with functional fitness in healthy elderly men: A pilot study. J. Exerc. Nutr. Biochem..

[19] Walters K., Frost R., Kharicha K., Avgerinou C., Gardner B., Ricciardi F., Hunter R., Liljas A., Manthorpe J., Drennan V. (2017). Home-based health promotion for older people with mild frailty: The homehealth intervention development and feasibility rct. Health Technol. Assess..

[20] Doody R.S., Ferris S.H., Salloway S., Sun Y., Goldman R., Watkins W.E., Xu Y., Murthy A.K. (2009). Donepezil treatment of patients with mci: A 48-week randomized, placebo-controlled trial. Neurology.

[21] Choi S.H., Shim Y.S., Ryu S.H., Ryu H.J., Lee D.W., Lee J.Y., Jeong J.H., Han S.H. (2011). Validation of the literacy independent cognitive assessment. Int. Psychogeriatr..

[22] Bae J.N., Cho M.J. (2004). Development of the korean version of the geriatric depression scale and its short form among elderly psychiatric patients. J. Psychosom. Res..

[23] Guralnik J.M., Simonsick E.M., Ferrucci L., Glynn R.J., Berkman L.F., Blazer D.G., Scherr P.A., Wallace R.B. (1994). A short physical performance battery assessing lower extremity function: Association with self-reported disability and prediction of mortality and nursing home admission. J. Gerontol..

[24] Morris J.C. (1993). The clinical dementia rating (cdr): Current version and scoring rules. Neurology.

[25] Suttanon P., Hill K.D., Said C.M., Williams S.B., Byrne K.N., LoGiudice D., Lautenschlager N.T., Dodd K.J. (2013). Feasibility, safety and preliminary evidence of the effectiveness of a home-based exercise programme for older people with alzheimer’s disease: A pilot randomized controlled trial. Clin. Rehabil..

[26] Kivipelto M., Mangialasche F., Ngandu T.J.N.R.N. (2018). Lifestyle interventions to prevent cognitive impairment, dementia and alzheimer disease. Nat. Rev. Neurol..

[27] Kivipelto M., Ngandu T., Laatikainen T., Winblad B., Soininen H., Tuomilehto J. (2006). Risk score for the prediction of dementia risk in 20 years among middle aged people: A longitudinal, population-based study. Lancet Neurol..

[28] Rosenberg A., Ngandu T., Rusanen M., Antikainen R., Bäckman L., Havulinna S., Hänninen T., Laatikainen T., Lehtisalo J., Levälahti E. (2018). Multidomain lifestyle intervention benefits a large elderly population at risk for cognitive decline and dementia regardless of baseline characteristics: The finger trial. Alzheimer’s & Dementia.

